# On the Larval Sensory Organs and Central Nervous System of the Zoea 1 of the Asian Shore Crab *Hemigrapsus sanguineus* (Decapoda, Brachyura)

**DOI:** 10.1002/cne.70201

**Published:** 2026-09-24

**Authors:** Steffen Harzsch, Johanna Blatt, Johanna Seegel‐Schultz, Lisa Riehemann, Noé Espinosa‐Novo, Jan Phillipp Geißel, Joshua Gauweiler, Alexandre Casadei‐Ferreira, Sebastian Büsse, Birk Rillich, Wolfgang Stein, Gabriela Torres, Roland Melzer

**Affiliations:** ^1^ Department of Cytology and Evolutionary Biology Zoological Institute and Museum, University of Greifswald Greifswald Germany; ^2^ Biologische Anstalt Helgoland Alfred‐Wegener‐Institut, Helmholtz‐Zentrum für Polar‐ und Meeresforschung Helgoland Germany; ^3^ Marine Biology Research Group Ghent University, Campus Sterre Ghent Belgium; ^4^ Instituto de Ciencias Oceánicas Universidad de la República Montevideo Uruguay; ^5^ Research and Collection Centre, Scientific Collections State Museums of Tyrol Hall in Tirol Austria; ^6^ Institut für Biowissenschaften Allgemeine & Spezielle Zoologie Universität Rostock Rostock Germany; ^7^ School of Biological Sciences Illinois State University Normal Illinois USA; ^8^ Bavarian State Collection of Zoology—SNSB Munich Germany; ^9^ Department of Biology II LMU Munich, Biocenter Planegg Germany; ^10^ GeoBio‐Center der LMU München Munich Germany

**Keywords:** aesthetascs, central body, complex life cycle, compound eye, lamina, lobula, medulla, olfactory lobe, organ of Bellonci, protocerebrum

## Abstract

Many decapod crustaceans occupy different habitats during their ontogeny and undergo a niche shift during the transition from the larval to the juvenile phase. Their life cycle comprises pelagic larvae and, in most species, benthic juvenile‐adult stages. Already at hatching, decapod larvae possess a wealth of organ systems, necessary to autonomously survive and develop in the plankton. They also exhibit a rich behavioral repertoire to appropriately respond to environmental stimuli such as light, gravity, hydrostatic pressure, tidal currents, temperature, salinity, chemical cues, and food concentration. Considering the impressive larval behavioral performance, neuroanatomists in the past have already studied selected aspects of the functional morphology of larval nervous systems, but this knowledge is distributed across a wide taxonomic range. Our study aims to provide a concise description of the morphology of selected sensory organs and that of the central nervous system in the Zoea 1 of the Asian shore crab *Hemigrapsus sanguineus* using multiple neuroanatomical methods such as three‐dimensional (3D) reconstruction from histological section series, scanning electron microscopy, and immunohistochemistry combined with confocal laser‐scan microscopy. The larval developmental cycle of this includes five zoea stages and one megalopa stage. In the Zoea 1, our data reveal well‐developed visual and olfactory organs comprising hundreds of sensory receptor neurons and also complex primary visual and olfactory neuropils related to processing this sensory input. We observed a particularly high investment of nervous tissue in the central visual pathway. Furthermore, the larval brain comprises several characteristic secondary neuropils which, as higher order neuropils, in the adult crustacean brain are thought to integrate highly processed multimodal sensory input. These are, for example, the lateral protocerebrum (which in adult crabs will house the mushroom bodies) and the median protocerebrum that includes the central body. The discussion of our findings is framed against the knowledge of the larval behavioral performance and, considering the larval investment in higher order neuropils, the possibility of complex behavioral decisions that integrate aspects of learning and memory.

## Graphical abstract

We provide a description of the morphology of selected sensory organs and of the central nervous system in the Zoea 1 of the Asian shore crab *Hemigrapsus sanguineus* using multiple neuroanatomical methods. Our data reveal well‐developed visual and olfactory organs and complex primary sensory neuropils related to processing this input.

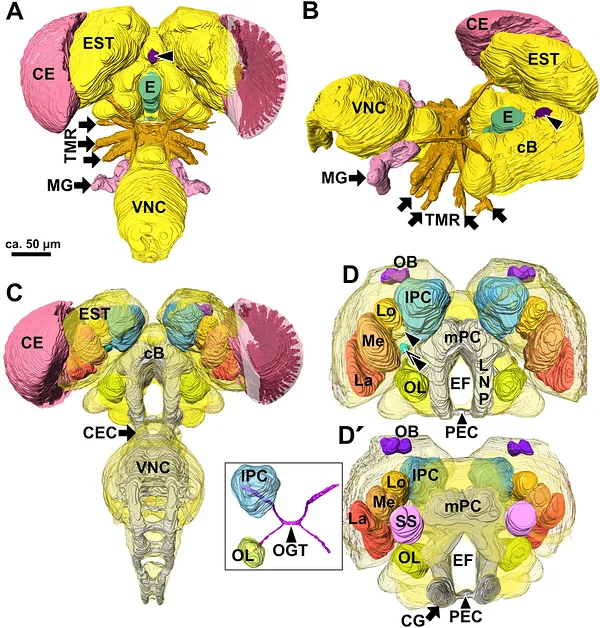

## 1 Introduction

“Larval crustaceans are highly adapted to their habitat—they are not just simple versions of the adults hanging around in the plankton waiting to metamorphose.” (Cronin et al. 2017).

As with many other marine benthic animals, decapod crustaceans typically exhibit a complex life cycle, in which they occupy different habitats during their ontogeny and undergo a niche shift during the transition from the larval to the juvenile phase. Their life cycle comprises pelagic larvae and, in most species, benthic juvenile‐adult stages (reviews, e.g., Rice 1980; Williamson 1982; Ingle 1992; Anger 2001, 2006; Martin et al. 2014; Anger et al. 2015; Jirikowski et al. 2015; Olesen 2018; Möller et al. 2020). In the pelagic larval phase, which is essential for dispersal (reviews, e.g., Cronin and Forward 1986; Morgan 2020), the larvae actively feed (review Jeffs and O'Rorke 2020) and grow through a species‐specific number of successive molts, which may vary depending on environmental conditions (reviews Haug and Haug 2015; Olesen 2018; Giménez 2020; Zeng et al. 2020). In the absence of parental care, morphological and behavioral traits related to locomotion, feeding, and sensory perception have enabled the larvae to adapt to developing in the plankton (reviews Anger 2001, 2006). In many decapod groups, larvae eventually settle on the seafloor. To ontogenetically bridge the large morphological and behavioral differences between larvae and later stages, these animals undergo a drastic double metamorphosis during this transition (reviews, e.g., Forward et al. 2001; Forward 2009; Gebauer et al. 2020; Haug 2020).

Already at hatching, decapod larvae possess a wealth of organ systems necessary to autonomously survive and develop in the plankton, and these organs persist into adulthood (reviews Rice 1980; Williamson 1982; Ingle 1992; Anger 2001; and Spitzner et al. 2018; Melzer et al. 2021). Decapod larvae also exhibit a rich behavioral repertoire, suggesting they can appropriately respond to environmental stimuli such as light, gravity, hydrostatic pressure, tidal currents, temperature, salinity, chemical cues, and food concentration (reviews Cohen and Forward 2009; Forward et al. 2001; Forward 2009; Epifanio and Cohen 2016; Epifanio 2013; Cohen and Epifanio 2020; Morgan 2020). Gravity, hydrostatic pressure, and light are considered the three central external cues affecting larval behavior, the former two stimuli being highly predictable (Epifanio and Cohen 2016). Underwater light is less predictable because its intensity, spectral composition, angular distribution, and polarization fluctuate with daytime and weather conditions. These factors also depend on water depth and concentration of dissolved particles (review, e.g., Cronin et al. 2014). Behavioral patterns related to the tidal rhythm, such as active vertical migration in response to tidal currents, enable the larvae to avoid predators and regulate their horizontal dispersal (reviews Cronin and Forward 1986; Forward 1998; Cohen and Forward 2009; Epifanio and Cohen 2016; Epifanio 2013; Morgan 2020). Furthermore, it is well known that later larval stages can perceive the chemical signatures of coastal waters, including olfactory cues from conspecifics to identify suitable habitats for the initiation of metamorphosis (reviews Forward et al. 2001; Forward 2009; Epifanio and Cohen 2016; Gebauer et al. 2020).

The Asian shore crab *Hemigrapsus sanguineus* is native to the east coast of Asia (20° to 50°N), where it is among the most abundant crab species in the upper and middle intertidal zones of rocky coasts. This species has become invasive along the coasts of North America, North Europe, and the Adriatic and Black Seas (reviews, e.g., Schubart 2003; Dauvin et al. 2009; Epifanio 2013; Karlsson et al. 2019; Bader et al. 2024). Both in northern America and in northern Europe, *H. sanguineus* currently expands its range further North (e.g., Jungblut et al. 2017; Geburzi et al. 2018; Espinosa‐Novo et al. 2023; Griffen et al. 2025), a process that is likely to be further fueled by ocean warming (Giménez et al. 2020). The nervous system morphology in adult representatives of the genus *Hemigrapsus* has previously been characterized. For example, the neuroanatomy of the central brain (Tsvileneva and Titova 1985; Tsvileneva et al. 1985) and of the lateral protocerebrum (Strausfeld and Sayre 2021), the neuroanatomy of the central visual pathway (Sztarker et al. 2009), the localization of neuroactive substances (Kotsyuba 2012; Kotsyuba and Dyachuk 2021), the structure of, and the circadian pigment migration within, the compound eyes (Arikawa et al. 1987), and their growth (Eguchi et al. 1989), have been studied. On the contrary, there does not exist any report on the larval nervous system in this species.

**FIGURE 1:**
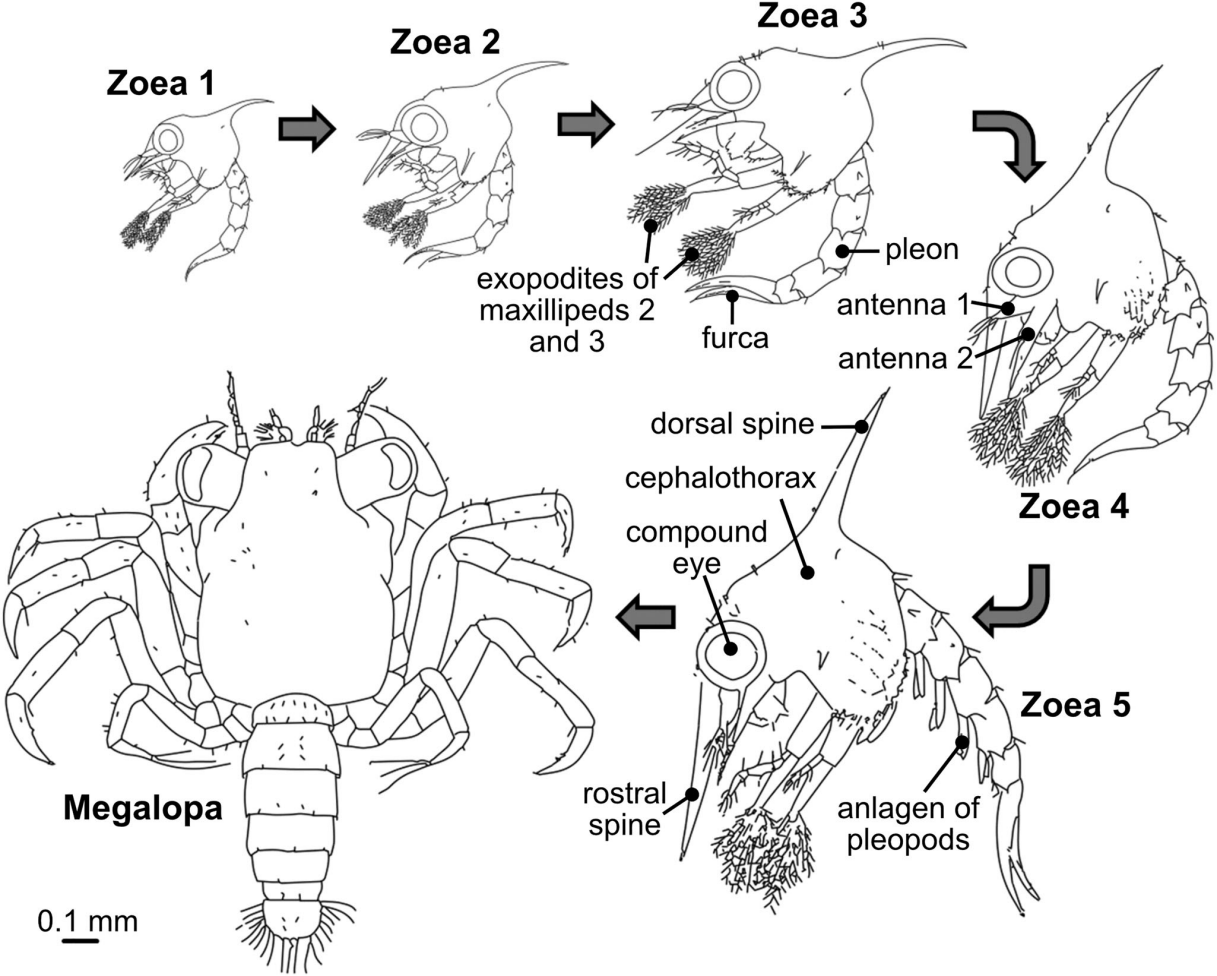
Larval development of *Hemigrapsus sanguineus* (modified from Hwang et al. 1993).

The larval developmental cycle of *H. sanguineus* includes five zoea stages and one megalopa stage as determined by laboratory culture (Figure 1; Hwang et al. 1993; Kornienko et al. 2008), and precise larval descriptions are available, specifically of the Zoea 1 (Lee and Ko 2008). Under environmental stress, for example, low temperature or a combination of moderate temperature and food limitation or low salinity, an additional sixth zoea is observed (Espinosa‐Novo et al. 2023). As is typical for the Zoea 1 of Brachyura (Martin et al. 2014), in *H. sanguineus* this stage is equipped with well‐developed—yet unstalked—compound eyes followed by antennae 1 and 2 as cephalic appendages. Adjoining posteriorly, the segments of the mandible, maxillae 1, maxilla 2 follow, equiped with appendages that function in the context of feeding. The larvae swim in the water column using the exopodites of maxilliped 1 and 2. Although in the Zoea 1, at hatching, all body segments that characterize the brachyuran Bauplan are established, the appendages maxilliped 3, all pereiopods, and all pleopods are not yet present at this stage and will gradually develop in the successive zoea stages (Figure 1; and Kornienko et al. 2008; Lee and Ko 2008).

In *H. sanguineus*, the effects of gravity and pressure on swimming behavior in newly hatched larvae have been analyzed (Park et al. 2004; Cohen et al. 2015), and the role of chemical cues in inducing metamorphosis in later larval stages has been examined (Anderson and Epifanio 2009; Anderson et al. 2010; Anderson and Epifanio 2010). Considering the larval behavioral performance, this study sets out to analyze the morphology of selected sensory organs and that of the central nervous system in the Zoea 1 of *H. sanguineus*, as this species rapidly expands its range so that it is readily accessible in German coasts. In the past, neuroanatomists have already studied selected aspects of the functional morphology of *larval* nervous systems in a wide range of different decapod crustaceans so that our knowledge of larval neuroanatomy is dispersed across a wide taxonomic range (see Table 1; for studies on aspects of *embryonic* organogenesis in decapod crustaceans, we refer the reader to the following selected book chapters: Scholtz and Wolf 2013; Wolff and Gerberding 2015; Loose et al. 2020). The larval sensory systems include compound eyes and a variety of chemo‐ and mechanosensory sensilla, whose input is processed by a sophisticated central nervous system that displays a diverse neurochemistry. In contrast, our knowledge of larval temperature, gravity, or pressure receptors is very limited (review Epifanio and Cohen 2016). Our study aims to provide a concise description of selected sensory organs and of the associated central nervous system in just one species using three‐dimensional (3D) reconstruction from histological section series, scanning electron microscopy, and immunohistochemistry combined with confocal laser‐scan microscopy.

**TABLE 1:** Studies on the larval nervous system in representatives of Brachyura.

**General neuroanatomy of the central nervous system and neurogenesis**
*Carcinus maenas* Harzsch and Dawirs (1993), Spitzner et al. (2018)
*Portunus trituberculatus* Nakamura (1990)
*Rhithropanopeus harrisii* Frydel (1975)
*Pachygrapsus marmoratus* Geiselbrecht and Melzer (2013)
*Cancer anthonyi* Trask (1974)
*Maja brachydactyla* Castejón et al. (2018)
*Hyas araneus* Harzsch and Dawirs (1994, 1996a), Harzsch et al. (1998)
**Neuroactive substances in the central nervous system**
C*. maenas* molt inhibiting hormone, crustacean cardioactive peptide, crustacean hyperglycemic hormone: Webster and Dircksen (1991), Chung and Webster (2004)
*H. araneus* serotonin: Harzsch and Dawirs (1995), RFamide: Harzsch and Dawirs (1996)
**Neuroendocrine centers**
*C. anthonyi* McConaugha (1980)
**Structure of compound eyes and eyeshine**
*Callinectes sapidus* Cronin et al. (1995)
*C. maenas* Harzsch and Dawirs (1995/96)
*H. araneus* Harzsch and Dawirs (1995/96)
*Hemigrapsus sanguineus* Charpentier and Cohen (2015)
*R. harrisii* Charpentier and Cohen (2015)
*Eyeshine* Cronin et al. (2017), Shavit et al. (2023)
**Various sensory setae**
*C. maenas* Ekerholm and Hallberg (2002), Spitzner et al. (2018)
*Portunus acuminatus* Meyer et al. (2006)
*Dissodactylus crinitichelis* Pohle and Telford (1981)
**Sensory dorsal organs**
*Various* Lerosey‐Aubril and Meyer (2013)

## 2 Materials and Methods

### 2.1 Sample Collection and Geographic Information

Ovigerous females of *H. sanguineus* (carapace widths 16.3–21.6 mm) were hand‐collected during low tide in the intertidal zone on the rocky shores of the island of Helgoland (54°10′57″ N, 7°53′7″ E), German Bight, North Sea, during a single reproductive period (2024). After collection, females were transported to the Biologische Anstalt Helgoland at the Alfred‐Wegener‐Institute, Helgoland, Germany (for reference, see Dummermuth et al. 2023) in natural seawater from the collection site. Only ovigerous females with early‐stage eggs were selected, and only larvae that hatched within 10–14 days of collection were usedto obtain samples for this study. Females were incubated in individual aquaria provided with refugia and natural UV‐treated, filtered (mesh size: 2 µm) seawater (salinity: 32.5 PSU) at constant temperatures (24°C) until larvae hatched. Females were fed twice weekly with locally sourced frozen shrimps (*Crangon crangon*), and water was changed daily to ensure high water quality at hatching. After hatching, randomly selected larvae were transferred to rearing units (250 mL crystallization dishes) at an initial density of 35 larvae per dish (0.14 ind/mL). Larval rearing was conducted at 21°C in temperature‐controlled rooms (±1°C) with a 12:12 h light:dark cycle for 24 h after hatching in seawater with ad libitum food (freshly hatched *Artemia* sp. nauplii—Great Salt Lake Artemia) following a standard protocol (Torres et al. 2021).

### 2.2 Classical Histology

The larvae were sampled for chemical fixation 24 h after hatching. Animals were immobilized and anesthetized by chilling on ice and then processed as described by Torres et al. (2021) and Melzer et al. (2021). Before immersion in the fixatives, the pleon was dissected to improve the penetration of the chemicals. For classical histology, specimens were incubated in the fixative following Karnovsky (1965); (2% paraformaldehyde, 2.5% glutaraldehyde, 5% glucose in 0.1 M phosphate buffer, and pH 7.1) and stored for further analysis. Specimens were then washed three times in sodium phosphate buffer (0.1 M, pH 7.4) for 10 min each. Subsequently, specimens were incubated in 2% osmium tetroxide for 4 h at room temperature for secondary fixation and washed with distilled water. Then, specimens were dehydrated in an ascending ethanol series (30%, 50%, 60%, 70%, 80%, 90%, and 96%; 15 min in each step) and washed three times with 100% ethanol at room temperature, then washed twice for 15 min with propylene oxide and infiltrated in an ascending mixture of propylene oxide:Embed812 (2:1, 1:1, and 1:2). Finally, specimens were embedded in Embed812 resin blocks (Electron Microscope Service). To remove air bubbles from the resin blocks, a Heraeus VT‐6025 vacuum heating cabinet was used at 150 mbar and 40°C for three 30‐min cycles, with 10‐min breaks at atmospheric pressure. Polymerization of the resin was carried out at 60°C for 40 h. Sections (1.5 µm thickness) were cut with the ZEISS Thermo Scientific Microm HM360 microtome and were stained with 1% phenylene‐diamine (dissolved in 50 mL methanol and 50 mL 2‐propanol; Holländer and Vaaland (1968); also see Harzsch and Dawirs (1993)), a reagent that strongly binds to phospholipids (Shirai et al. 2016). The sections were incubated in the staining solution overnight at room temperature with gentle agitation, then differentiated twice for 30 min in 2‐propanol. The sections were cover‐slipped using the ROTI Histokit II (Roth). Light microscopic images of single sections were taken with a Nikon Eclipse 80i. Images were assembled in plates using Affinity Designer 2.

### 2.3 Digitizing Section and Images, 3D Reconstruction, and Visualization

The histological sections were digitized using an Axioscan 7 slide scanner (Zeiss) at the University of Rostock at a magnification of 20×. The slide scanner enabled automated, uniform illumination of the slides as well as automatic stitching of the manually selected areas. Prior to alignment, image processing was performed using the software IrfanView 64 4.70 (by Irfan Škiljan). Through batch conversion, the images were centered on a canvas scaled to match the largest image in the batch, ensuring that all images had the same dimensions. To enable automatic alignment, the background color of the canvas was matched to the background color of the images.

3D reconstructions of the central nervous system were generated from the digitized images of a frontal section series (136 sections, 1.5 µm thickness) using the aligning tool and the segmentation manager editor in Amira 6 with the help of a drawing pad. 3D visualizations were performed using the surface generation and visualization tools in Amira.

### 2.4 Reconstruction and Quantification of Compound Eye Ommatidia

Aligned image stacks were imported as TIFF files into 3D Slicer v5.8.1 (Brigham and Women's Hospital, USA) (Fedorov et al. 2012) to generate 3D reconstructions of individual ommatidia. Ommatidial number was quantified by counting the total number of individual segmentation labels in the final reconstructed volume. Volume renderings for figure preparation were generated in 3D Slicer using the *Colorize Volume* module available in the *SlicerSandbox* extension.

### 2.5 Scanning Electron Microscopy

The larvae were processed for scanning electron microscopy as we have previously described in Melzer et al. (2021). Briefly, the larvae were chemically fixed and osmicated as described above in the section on “classical histology.” The larvae were then dehydrated in a graded series of ethanol (concentrations 10%, 30%, 50%, 70%, 90%, and 100%) with 20 min per step at room temperature. The specimens were kept in 100% ethanol until they were critical point dried using a critical point dryer Leica EM CPD300 (Leica Microsystems) and then mounted on aluminum stubs. Samples were sputtered with a Sputter coater Polaron SC7640 (Fisons Instruments/Quorum) and examined with a Zeiss EVO LS10 (Carl Zeiss Microscopy, Deutschland).

### 2.6 Immunohistochemistry and Confocal Laser‐Scanning Microscopy

For immunohistochemistry, animals were immobilized and anesthetized by chilling on ice and then processed as described by Torres et al. (2021) and Melzer et al. (2021). Specimens were fixed overnight in 4% paraformaldehyde in 0.1 M phosphate buffered saline. Before immersion in the fixatives, the pleon was dissected to improve the penetration of the chemicals. Then, specimens were washed twice for 10 min in phosphate buffered saline (PBS; pH 7,4, 0.1 M) and infiltrated for 20 min in 1:1 mixture of cryoprotectant:PBS to be finally stored in cryoprotectant for further analysis (a mixture 50:30:1:30 of PBS:sucrose:polyvinylpyrrolidone:ethylene glycol) at −20°C. For immunohistochemical experiments, the larval brains were manually dissected using micro forceps (see Torres et al. 2021), then washed in several changes of PBS for 2 h at room temperature and blocked with 1% bovine serum albumin (BSA, Sigma, A5253‐250G, lot SLBL7392V) diluted in PBS with 0.3% Triton X‐100 (PBS‐TX, Sigma‐Aldrich, X100 500ML, lot STBJ5677) twice for 15 min, followed by 2 × 30 min incubation in this reagent at room temperature. Subsequently, the primary antisera were added.

The following mixtures of antisera were utilized: anti‐synapsin SYNORF1, raised in mouse (dilution 1:10 in PBS‐TX, Developmental Studies Hybridoma Bank #3C11, RRID AB_528479) combined with either anti‐Serotonin (dilution 1:1000, Immunostar, #20080, raised in rabbit, RRID AB_572263), or anti‐FMRFamide (dilution 1:2000, Immunostar, #20091, raised in rabbit, RRID AB_572232), or anti‐SIFamide (dilution 1:1500 in PBS‐TX, raised in rabbit, Janssen et al. (1996), RRID AB_2569992), or anti‐Asn13‐orcokinin (Asn13‐OK) (dilution 1:2000; Bungart et al. (1994); Dircksen et al. (2000); raised in rabbit, AB_2315017), or anti‐A‐type Dip allatostatin I (dilution 1:2000, Jena Bioscience, abd‐062; RRID: AB_2314318). The individual markers were chosen because well‐established protocols were available, which were easy to adapt, and because preliminary results had indicated successful labeling with the specific antisera similar to that seen in the developing (review Harzsch and Viertel 2020) and adult (Harzsch et al. 2022) central nervous system of decapod crustaceans. Furthermore, some of these markers were already used for analyzing the crab larval nervous system (Harzsch and Dawirs 1995; Harzsch and Dawirs 1996b) so that these studies can serve as a basis for comparison.

Specimens were incubated in the primary antisera for between 1 and 3 days. Then, tissues were rinsed with PBS‐TX twice for 10 min, twice for 20 min before the mixture of secondary antisera was added (1:500 goat anti‐mouse conjugated to Cyanine 3, Jackson Immuno Research, RRID: AB_2338000; and 1:500 goat anti‐rabbit conjugated to Alexa Flour 488, Invitrogen, Thermo Fischer Scientific, RRID: AB_10374301). Furthermore, a nuclear marker (1:10,000, Hoechst 33342) was used in some samples for counter‐staining of the nuclei. Secondary antisera were incubated at room temperature in the dark for 2 days with gentle agitation. After washing with PBS twice for 10 min, twice for 20 min, and again for at least 1 h, tissues were infiltrated with 1:1 mixture of PBS:glycerol for 60 min, followed by 30 min infiltration with a 1:3 or 1:9 PBS:glycerol solution. Thereafter, tissues were embedded in 8 µL Dabco‐glycerol solution (200 µL distilled water, 50 mg Dabco, 800 µL glycerol; Carl Roth, lot 294216515).

In control experiments, we replaced the primary antisera with phosphate buffered saline in which case all staining was abolished. Immunohistochemical preparations were scanned using a Leica TCS SP5 II confocal laser‐scanning microscope equipped with Argon‐, DPSS‐, and Diode‐lasers and operated with the Leica Application Suite Advanced Fluorescence software package (LAS AF). For the analysis, at least ten successfully stained and imaged specimens were used per marker set. Single optical sections, as well as z‐projections, were globally enhanced in ImageJ (Version 2.9.0, Schindelin et al. 2012).

### 2.7 Specificity of the Antisera

#### 2.7.1 Synapsins

The monoclonal anti‐*Drosophila* synapsin SYRNOF1 antibody (Developmental Hybridoma Bank (DSHB) Hybridoma Product 3C11, anti‐SYRNOF1 as deposited to the DSHB by E. Buchner, University Hospital Würzburg, Germany; supernatant) was raised against *Drosophila melanogaster* GST‐synapsin fusion protein and recognizes at least four different synapsin isoforms (70, 74, 80, and 143 kDa) in western blots of *D. melanogaster* head homogenates (Klagges et al. 1996). *D. melanogaster* *Coenobita clypeatus*, (Harzsch and Hansson 2008). Sullivan et al. (2007) Harzsch and Hansson (2008) conducted a western blot analysis comparing brain tissue of *D. melanogaster* and the hermit crab *C. clypeatus* (Anomura, Coenobitidae). The SYNORF1 serum provided identical results for both species, and it stained one strong band between 80 and 90 kDa and a second weaker band slightly above 148 kDa, suggesting that the epitope that SYNORF1 recognizes is strongly conserved between *D. melanogaster* and *C. clypeatus* (see Harzsch and Hansson 2008). The antibody was used to study, for example, the developing (Harzsch et al. 1998, 1999) and adult nervous system in brachyuran crabs (Krieger et al. 2012). It provides a labeling pattern that is consistent with the assumption that this reagent binds to synaptic neuropils in decapod crustaceans. Because no further experiments to test for the specificity of this antiserum in *H. sanguineus* were conducted, we will refer to the labeling as “synapsin‐like immunoreactivity.”

#### 2.7.2 Serotonin

We used an antiserum against serotonin that is a polyclonal rabbit antiserum raised against serotonin coupled to BSA with paraformaldehyde. The antiserum was quality control tested by the manufacturer using standard immunohistochemical methods. According to the manufacturer, staining with the antiserum was completely eliminated by pretreatment of the diluted antibody with 25 µg of serotonin coupled to BSA per mL of the diluted antibody. Harzsch et al. (2022), working on the nervous system of a hermit crab, repeated this control with the serotonin‐BSA conjugate that was used for generation of the antiserum as provided by ImmunoStar (Cat. No. 20081, Lot No. 750256; 50 µg of lyophilized serotonin creatinine sulfate coupled to BSA with paraformaldehyde). Preadsorption of the antibody in working dilution with the serotonin‐BSA conjugate at a final conjugate concentration of 10 µg/mL at 4°C for 24 h completely blocked all immunolabeling (Harzsch et al. 2022). The manufacturer also examined the cross reactivity of the antiserum. According to the data sheet, with 5, 10, and 25 µg amounts, the following substances did not react with the antiserum diluted to 1:20,000 using the horse radish peroxidase (HRP) labeling method: 5‐hydroxytryptophan, 5‐hydroxyindole‐3‐acetic acid, and dopamine.

#### 2.7.3 Allatostatins

The A‐type allatostatins (A‐ASTs; synonym Dip‐allatostatins) constitute a large family of neuropeptides that were first identified from the cockroach *Diploptera punctata* and that share the C‐terminal motif–YXFGLamide (reviews Nässel and Homberg 2006; Stay and Tobe 2007). In decapod crustaceans, almost 20 native A‐ASTs and related peptides were initially identified from extracts of the thoracic ganglia of the shore crab *Carcinus maenas* (Duve et al. 2002) and shortly after, several other A‐ASTs were isolated from the freshwater crayfish *Orconectes limosus* (Dircksen et al. 1999). Yasuda‐Kamatani and Yasuda (2006) have shown that more than 25 closely related ASTA‐like peptides occur on the same crayfish *Procambarus clarkii* allatostatin‐like peptide precursor, and could thus be co‐released. Meanwhile, the family of crustacean A‐ASTs has substantially grown to several dozens of representatives (review Christie et al. 2010), and Christie (2016) predicted a total of 29 peptides with the C‐terminal motif, ‐YXFGLamide, in a bioinformatic analysis on the peptidome of the shore crab *C. maenas*. In more recent studies, allatostatins were found in the cadiac ganglion of *Cancer borealis* (DeLaney and Li 2020) and within the neuropeptidome of the American lobster *Homarus americanus* (Lu et al. 2024).

We used an antiserum that was raised against the *D. punctata* (Pacific beetle cockroach) A‐type Dip‐allatostatin I, APSGAQRLYGFGLamide, coupled to bovine thyroglobulin using glutaraldehyde (Vitzthum et al. 1996) that has previously been used to localize A‐ASTs in malacostracan crustacean nervous systems (e.g., Dircksen et al. 1999; Utting et al. 2000; Harzsch and Hansson 2008; Polanska et al. 2012), including brachyuran crabs (Skiebe 1999; Krieger et al. 2012). Competitive ELISA with Dip‐allatostatin I, II, III, IV, and B2 showed that the antiserum is two orders of magnitudes more sensitive to Dip‐allatostatin I than to Dip‐allatostatins II, III, IV, and B2 (Vitzthum et al. 1996). Furthermore, the antiserum displays no crossreactivity with corazonin, CCAP, FMRFamide, leucomyosuppression, locustatachykinin 11, perisulfakinin, and proctolin as tested by noncompetitive ELISA. Preadsorption of the diluted antisera against Dip‐allatostatin I abolished all immunostaining in the brain sections of *Schistocerca gregaria* (Vitzthum et al. 1996). A sensitive competitive enzyme immunoassay (EIA) confirmed the high specificity of the antiserum for A‐type Dip‐allatostatin I (Dircksen et al. 1999). Because we did not further analyze the specificity of the antiserum in *H. sanguineus*, the term “allatostatin A‐like immunoreactivity” is used throughout this study.

#### 2.7.4 Orcokinins

Orcokinins represent a highly conserved family of neuropeptides the first members of which were discovered in extracts (Stangier et al. 1992) and then in neurons of the abdominal nerve cord of the crayfish *Orconectes limosus* (Astacida; Dircksen et al. 2000). Yasuda‐Kamatani and Yasuda (2000, 2006) identified two different orcokinin gene products in the crayfish *P. clarkii* showing that the cloned mRNA precursors gave rise to multiple copies of the first discovered and name‐giving Asn13‐OK but also to single copies of another four isoforms with modified C‐terminal or internal amino acids. In malacostracan crustaceans, these neuropeptides are widely distributed in the nervous system and display strong myotropic and neuromodulatory activities (Bungart et al. 1994, 1995; Dircksen et al. 2000; Li et al. 2002).

We used a rabbit anti‐Asn13‐OK antiserum (Bungart et al. 1994) that was raised against a glutaraldehyde‐conjugate of bovine thyroglobulin and Asn13‐OK. Most likely because of the very conserved N‐terminal sequence NFDEIDR in most orcokinins discovered to date, the Asn13‐OK‐antiserum showed almost full cross‐reaction with Val13‐orcokinin (Dircksen et al. 2000) and likely all other C‐terminally modified crustacean and even identified insect (cockroach *Rhyparobia* = *Leucophaea maderae*) orcokinins (Hofer et al. 2005). We have previously used this antiserum to label the brain of hermit crabs (Harzsch et al. 2022; Polanska et al. 2020). As we did not do any preadsorption experiments ourselves nor any analyses of this peptide family in *H. sanguineus*, we will refer to the labeling in our study as “orcokinin‐like immunoreactivity.”

#### 2.7.5 FMRFamide‐Related Peptides (FaRPs)

The tetrapeptide FMRFamide and FaRPs form a large neuropeptide family with more than 50 members all of which share the RFamide motif (Dockray 2004; Nässel and Homberg 2006; Zajac and Mollereau 2006). In malacostracan Crustacea, at least 12 FaRPs have been identified and sequenced from crabs, shrimps, lobsters, and crayfish (Huybrechts et al. 2003; Mercier et al. 2003), which range from seven to 12 amino acids in length and most of which share the carboxyterminal sequence Leu‐Arg‐Phe‐amide. The utilized antiserum was generated in rabbit against synthetic FMRFamide (Phe‐Met‐Arg‐Phe‐amide) conjugated to bovine thyroglobulin. According to the manufacturer, immunohistochemistry with this antiserum is completely eliminated by pretreatment of the diluted antibody with 100 µg/mL of FMRFamide. Harzsch and Hansson (2008) repeated this experiment in the anomuran *C. clypeatus* and preincubated the antiserum with 100 µg/mL FMRFamide (Sigma; 16 h, 4°C) resulting in a complete abolishment of all staining. We have previously used this antiserum to analyze brain structure in a variety of crustaceans (e.g., Polanska et al. 2012, 2020; Krieger et al. 2020; Kümmerlen et al. 2023, 2025; Harzsch et al. 2022), including several brachyuran crabs (Harzsch and Dawirs 1996b; Krieger et al. 2012, 2015). Because the crustacean FaRPs known so far all share the carboxyterminal sequence LRFamide, we conclude that the antiserum that we used most likely labels any peptide terminating with the sequence RFamide. Therefore, we will refer to the labeled structures in our specimens as “RFamide‐like immunoreactivity” throughout the study.

#### 2.7.6 SIFamides

SIFamides are a family of arthropod neuropeptides with a highly conserved sequence (review Verleyen et al. 2009) the first member of which was purified from grey flesh flies *Neobelliera bullata* (originally termed Neb‐LFamide; Janssen et al. 1996). Several isoforms were identified by extensive genome and peptidome analyses of many pancrustaceans, including several hexapods and also arachnids. All of them share the C‐terminal motif—SIFamide (reviewed in Verleyen et al. 2009). Crustacean‐SIFamide is a 1381‐Da peptide that has been identified in the crayfish *P. clarkii* (Yasuda and Yasuda‐Kamatani 2002; Yasuda et al. 2004) by topological mass spectrometry analysis in combination with MALDI‐TOF MS using slices of tissues, chromatographic purification from the extract of tissues, molecular cloning for the determination of the precursor structure, and capillary liquid chromatography tandem mass spectronemy (LC–MS/MS) analysis for elucidation of its posttranslational modifications. The cDNA of this peptide has been characterized to encode a 76 amino acid precursor protein that contains a signal sequence, one copy of GYRKPP FNGSIFG, and one additional peptide. Initial reverse transcriptase polymerase chain reaction (RT‐PCR) analysis had demonstrated the presence of mRNA of this neuropeptide throughout the crayfish brain. The antigen used for generation of the antiserum used in the present study (injection into rabbit) was (Cys)GYRKPPFNGSIF‐CONH2 conjugated to BSA by the material basis set (MBS) method with NH2 (Yasuda et al. 2004). These authors, and Yasuda‐Kamatani and Yasuda (2006) further characterized the antiserum and analyzed it for immunohistochemistry in crayfish. A digoxigenin‐labeled antisense RNA probe prepared by in vitro transcription with partial preprotein cDNA as a template (1‐313 in AB036713) was used for an in situ hybridization analysis of the crayfish brain (Yasuda‐Kamatani and Yasuda 2006). The crustacean‐SIFamide mRNA expression pattern was identical with the data obtained by the previous immunohistochemical analysis (Yasuda et al. 2004). The antiserum was also used to label the olfactory projection neurons in the crab *Libinia emarginata* (Sullivan and Beltz 2005), and another crayfish species (Polanska et al. 2007). Raspe et al. (2023) repeated a preadsorption experiment in an amphipod crustacean and expanded our knowledge on immunolocalization of this peptide beyond the decapod crustaceans. Because we do not have any information on the SIFamide in *H. sanguineus*, we will refer to the labeling in our study as “SIFamide‐like immunoreactivity.”

## 3 Results

**FIGURE 2:**
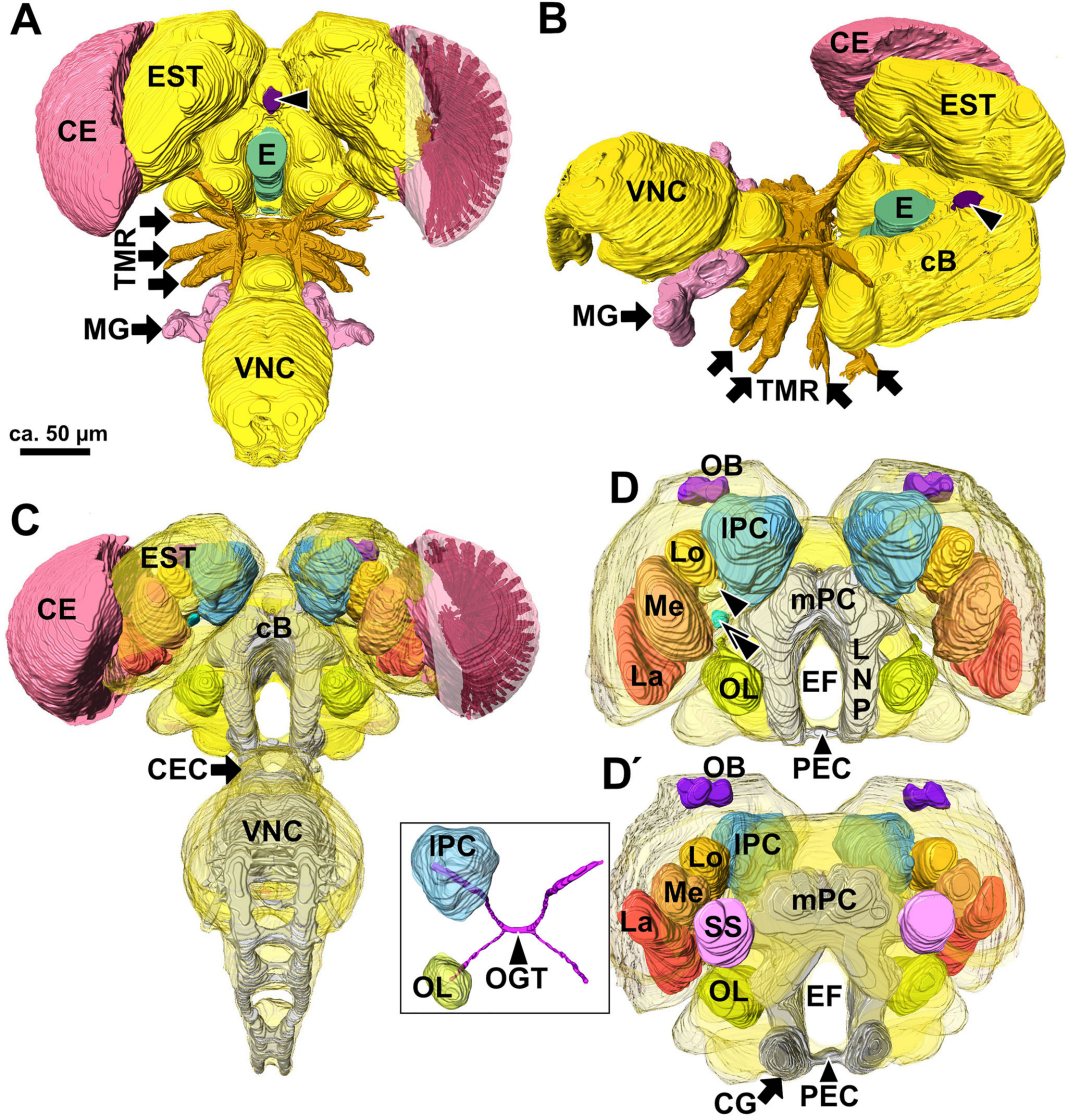
*Hemigrapsus sanguineus*, Zoea 1: reconstruction (Amira) of the nervous system from a horizontal section series (135 sections at 1.5 µm, stained with phenylene‐diamine). Anterior is to the top (A, C, and D) or to the right (B). (A and B) Dorsal (A) and dorsolateral (B) views of the compound eyes (magenta; reconstruction of the ommatidia in the right eye shown as an overlay in (A); right eye not shown in (B) and outer contour of the central nervous system (CNS; solid yellow). Arrowheads identify the cerebral artery. The position of the esophagus (green) that pierces the central brain is indicated, as is a conspicuous system of transverse muscles (TMR; orange) and the maxillary glands (MG; pink) in proximity to the nervous system. (C) same as in (B), but with a transparent outer contour of the central nervous system (light yellow) to show the neuropils within the CNS. Parts (D, D**′)** higher magnification of the eyestalk neuropils and central brain form a dorsal (D) and a ventral (D′) perspective. The single arrow in (D) identifies the satellite neuropil (see Figure 5B), and the double arrowhead identifies the sinus gland. **Inset**: the olfactory‐globular tract (purple) links the olfactory lobe to the lateral protocerebrum. Note that the scale bar in (A) is an approximation. cB, central brain; CE, compound eye; CEC, circumesophageal connective; CG, commissural ganglia; E, esophagus; EF, esophageal foramen; EST, ganglia in the developing eyestalks; La lamina, LNP longitudinal neuropil; Lo, lobula; lPC, lateral protocerebrum; Me, medulla; MG, maxillary gland; mPC, median protocerebrum; OB, organ of Bellonci; OGT, olfactory‐globular tract; OL, olfactory lobe; PEC, postesophageal commissure; SS, somata of sensory neurons; TMR, transverse muscle rigging associated with maxilla 1 and 2; VNC, ventral nerve cord

**FIGURE 3:**
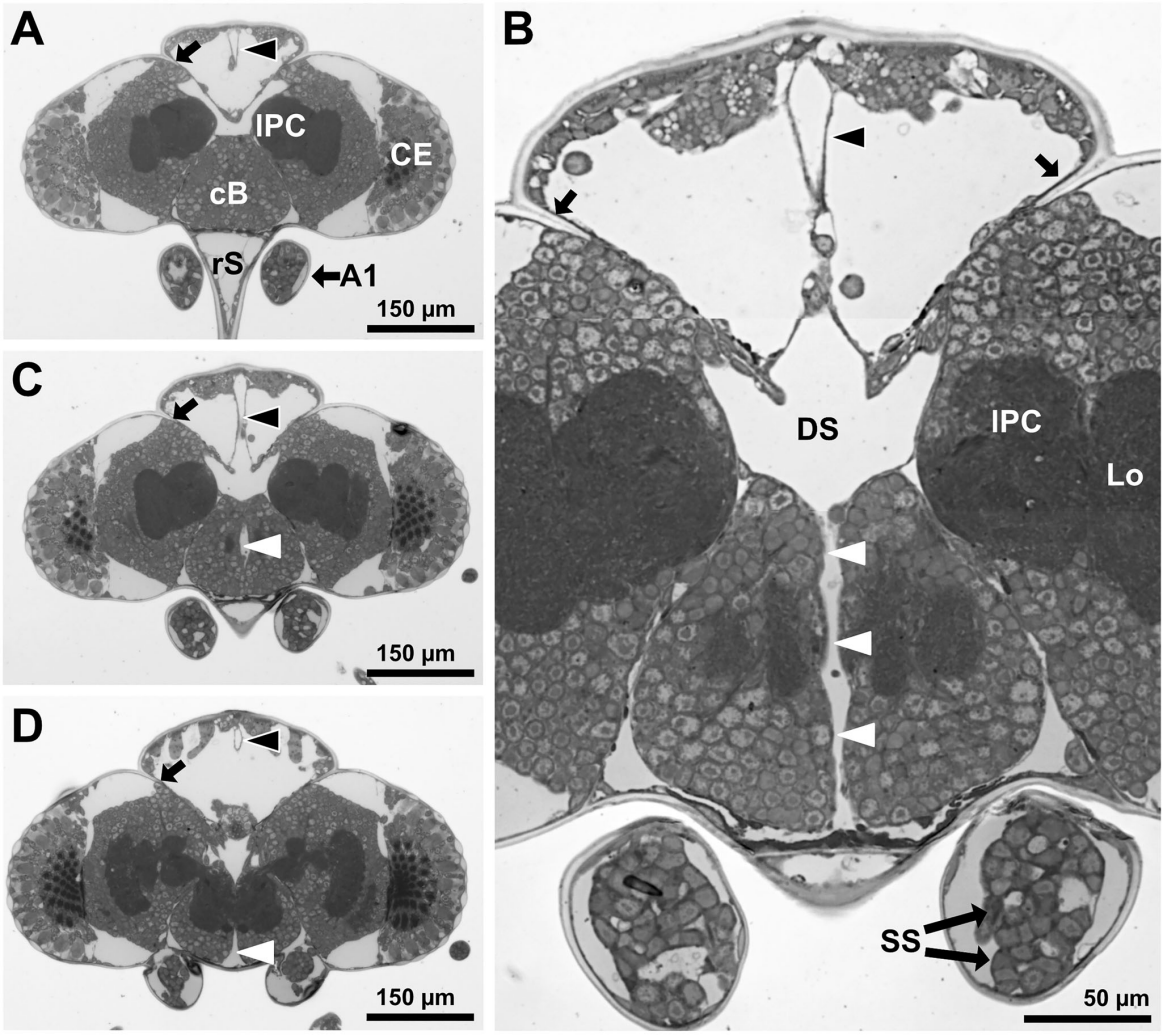
*Hemigrapsus sanguineus*, Zoea 1: selected frontal sections from an anterior (A)‐to‐posterior (C) series (1.5 µm, stained with phenylene‐diamine) showing various details of the brain. Black arrowheads identify the anterior aorta, and white arrowheads the cerebral artery, which originates from a dorsal sinus above the brain (D). The arrows identify cuticular extensions that prefigure the shape of the developing eyestalks. In (D), the somata of putative chemosensory neurons associated with the sensilla on the first pair of antennae are seen to be clustered in the proximal part of these appendages. A1, antenna 1; cB, central brain; CE, compound eye; DS, dorsal sinus; Lo, lobula; lPC, lateral protocerebrum; rS, rostral spine; SS, somata of sensory neurons.

As revealed by frontal and horizontal section series (e.g., Figures 3, 5, 7, and 8), the larval cephalothorax is tightly packed with various organ systems, but is dominated by the central nervous system, associated sensory organs, and the digestive system (cf., Spitzner et al. 2018). In a 3D reconstruction of a horizontal section series (Figure 2), the bilaterally paired compound eyes (CEs), their respective visual neuropils within the developing eyestalk (EST), as well as the central part of the brain (cB) are well recognizable. The latter is penetrated by the esophagus (E; Figures 2A,B and 7D). The central brain is caudally adjoined by the ventral nerve cord (VNC), a composite of the subesophageal, thoracic, and pleonal neuromeres. A conspicuous transverse system of muscle rigging (TMR; Figure 2A,B) associated with the first and second pair of maxillae is located just dorsal to the circumesophageal connectives (CECs) that link the central brain and the VNC. Slightly posteriorly, the VNC is flanked by the bilaterally paired maxillary glands (MG; Figures 2A,B and 8A).

### 3.1 Compound Eyes and the Central Visual Pathway

**FIGURE 4:**
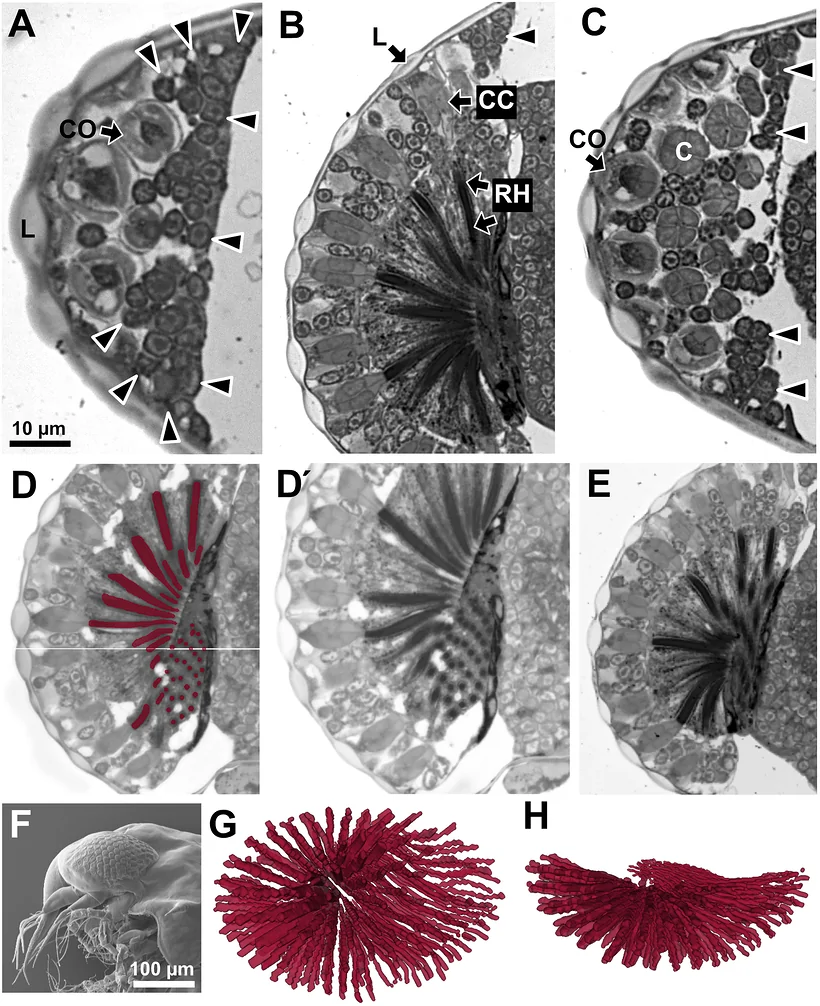
*Hemigrapsus sanguineus*, Zoea 1: selected sections from frontal (A–C and E) and horizontal (D) series (1.5 µm, stained with phenylene‐diamine) showing various details of the compound eyes. (A–C): Tangential and transverse sections reveal details of the ommatidial morphology. Arrowheads identify a band of undifferentiated cells at the rim of the ommatidial array, presumably the proliferation zone. (D, D′, E) Horizontal (D, D′) and frontal (C) sections across the center of the left eye reveal that the ommatidia (rhabdoms colored in red in D) do not display a perfect radial arrangement. (F) Scanning electron micrograph of the left side of the cephalothorax showing the sessile compound eye (anterior is towards the left). (G, H) Lateral (G) and dorsal (H) views of the left eye (anterior is to the left), reconstruction of the rhabdoms (red). Scale bar in (A) also applies for (B–E). C, crystalline cone; CC, crystalline cone cells; CO, corneagenous cell; L, corneal lens; RH, rhabdom.

In the Zoea 1, the CEs are not yet movable on eyestalks but are sessile (Figures 3A,C,D and 4F), as already noted by Lee and Ko (2008). Thin extensions of the cuticle (arrows in Figures 3B and 8A) prefigure the shape of the eyestalks that will become mobile during the molt to Zoea 2 (Hwang et al. 1993). Details of the ommatidial morphology are shown in Figure 4A–C. From proximal to distal, the corneal lens (L) associated with the corneagenous cells (CO), the crystalline cone (C), and associated crystalline cone cells (CC), and the rhabdom (RH) are visible. We also identified a band of undifferentiated cells at the rim of the ommatidial array (arrowheads in Figure 4A–C), resembling the proliferation zone that will contribute new ommatidia to the eye's margin during subsequent growth (see Harzsch and Dawirs 1995/96). Horizontal and frontal sections across the center of the left eye revealed that the ommatidia do not display a perfect radial arrangement (Figure 4D,D′,E; rhabdoms colored in red in D). Instead, in these sections, perfect tangential aspects but also cross sections of the rhabdom are visible, suggesting the ommatidia are arranged at a 90° angle in this zone. On the basis of reconstructions of the rhabdoms (Figure 4G,H), we counted 108 (left) and 110 (right) ommatidia within the CEs.

**FIGURE 5:**
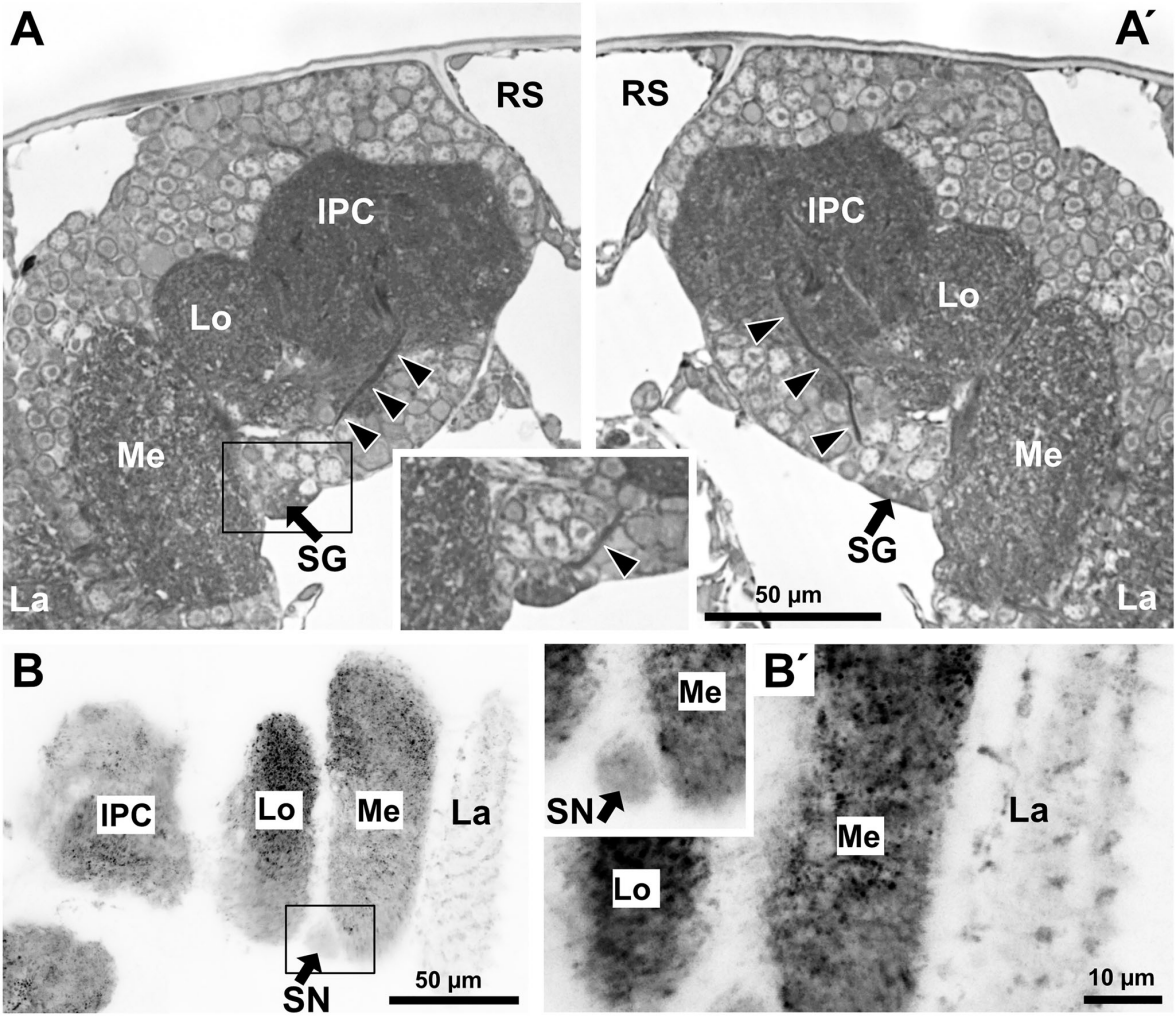
*Hemigrapsus sanguineus*, Zoea 1; (A, A′): horizontal sections (1.5 µm, stained with phenylene‐diamine) of the left (A) and right (A′) developing eyestalks, anterior is towards the top. Arrowheads identify a neurite bundle that extends from the lateral protocerebum to target the sinus gland. The **inset** is a higher magnification of the boxed area in (A). The arrow head points to a bundle of neurites targeting the sinus gland. (B) Immunohistochemical localization of synapsins in the visual neuropils of the right developing eyestalk (horizontal view, anterior is towards the top; black–white inverted confocal laser‐scan image). (B′) Higher magnification of (B) to show the sparse labeling within the lamina. The **inset** shows a higher magnification of the satellite neuropil. La, lamina; Lo, lobula; lPC, lateral protocerebrum; Me, medulla; RS, rostral sinus; SG, sinus gland; SN, satellite neuropil.

**FIGURE 6:**
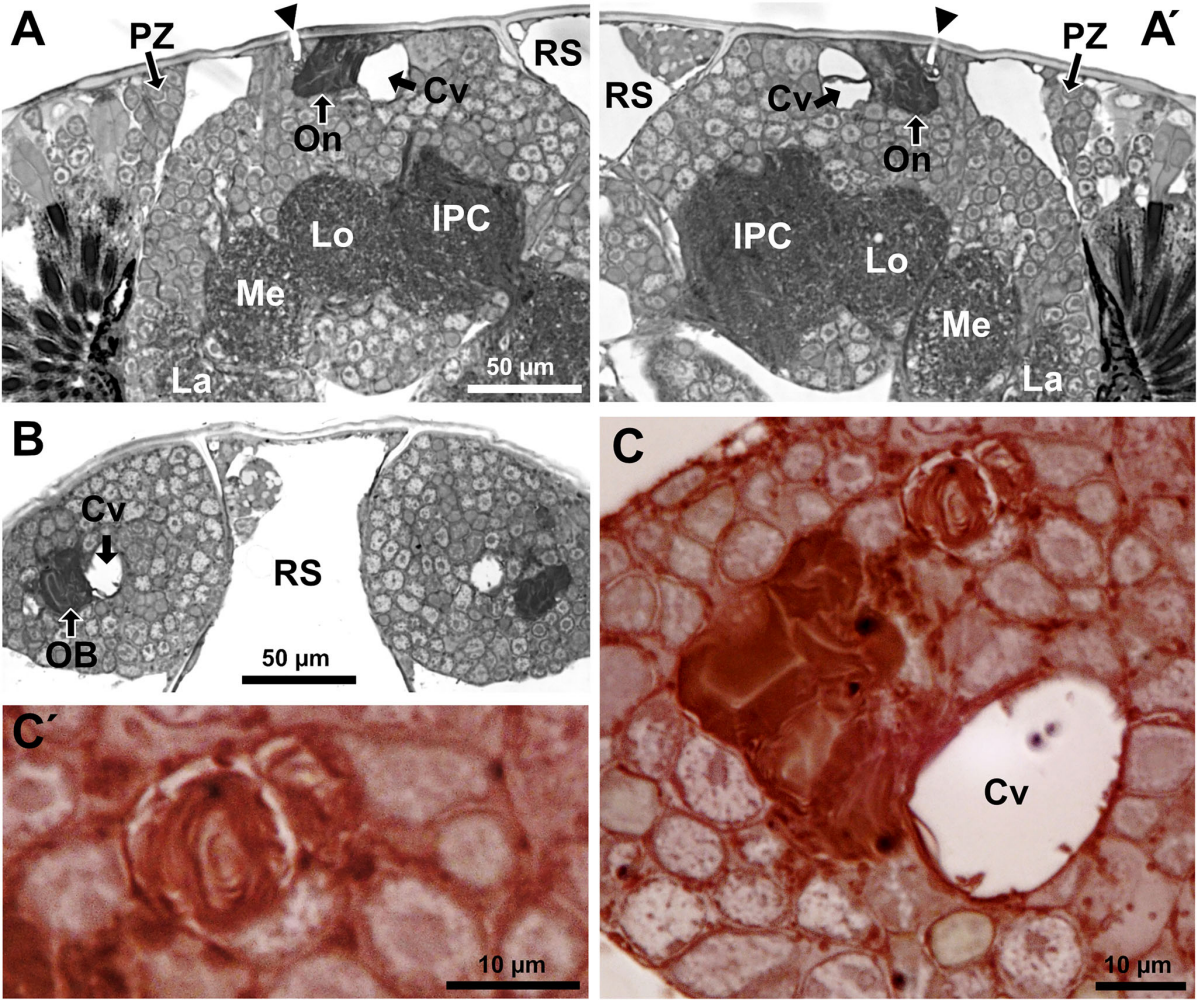
*Hemigrapsus sanguineus*, Zoea 1: selected horizontal (A, A′) and frontal (B, C, C′) sections (1.5 µm, stained with phenylene‐diamine) to show the organ of Bellonci. (A, A′) Sections of the left (A) and right (A′) developing eyestalk shows that the onion bodies are medially flanked by a cavity and anteriorly associated with a cuticular pore (arrowhead). (B) frontal section showing the symmetrical arrangement of the onion bodies and cavity in the left and right developing eyestalks. (C, C′) Higher magnifications of the onion bodies to show the concentric lamellar material. Cv, cavity; La, lamina; Lo, lobula; lPC, lateral protocerebrum; Me, medulla; On, onion body; PZ, proliferation zone at the margin of the compound eye; RS, rostral sinus.

From distal to proximal, each of the developing eyestalks houses the visual neuropils lamina (La), medulla (Me), and lobula (Lo; see Figure 2D), which are very prominent in frontal (Figure 3) and horizontal (Figures 5A and 6A) sections. The medulla and lobula were labeled with an antiserum against synaptic proteins (Figures 5B and 8F′). The histological sections (Figure 5A), and counterstaining with a DNA marker combined with confocal laser‐scan microscopic analysis (Figure 8F) revealed that a thick layer of neuronal somata surrounds the neuropils. These techniques also revealed the presence of a small spherical satellite neuropil (SN) located between the posterior tips of the medulla and lobula (single arrowhead in Figure 2D; inset in Figure 5B; arrowheads in Figures 8F′,F″ and 13A). Anti‐synapsin immunohistochemistry showed only sparse labeling within the lamina (Figure 5B,B′), but nevertheless, a regular pattern of the labeled profiles was visible (compare Harzsch et al. 1997). A similar finding was previously reported from the vinegar fly *D. melanogaster* in which not only the lamina but also a certain layer of the medulla showed only very weak staining with the anti‐synapsin antiserum that we used here (Klagges et al. 1996). These authors concluded that in *D. melanogaster*, photoreceptors Rl‐R6, which have their synapses in the lamina, and at least some of the cells with synaptic terminals in or near layer M5 of the medulla contain no or very little of the presently known synapsin homolog isoforms.

### 3.2 Lateral Protocerebrum, Sinus Gland, and Organ of Bellonci

**FIGURE 7:**
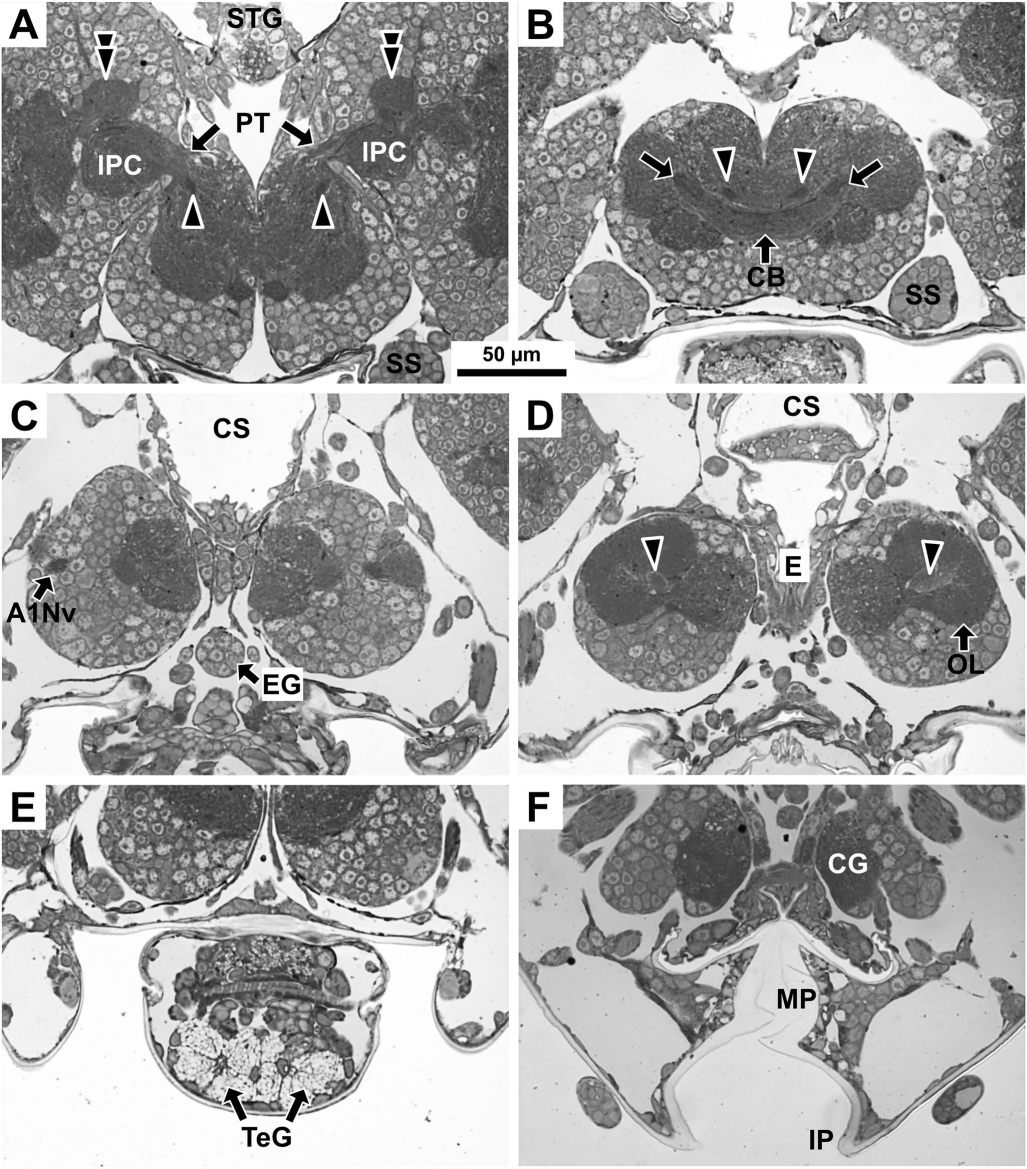
*Hemigrapsus sanguineus*, Zoea 1: selected frontal sections from an anterior (A)‐to‐posterior (F) series (1.5 µm, stained with phenylene‐diamine) showing various details of the brain. Single arrowheads: olfactory‐globular tract, double arrowheads: dome‐shaped neuropil, arrows (in B) identify the central body. The scale bar in (A and B) applies to all images of the plate. A1Nv, antenna 1 nerve; CB, central body; CG, commissural ganglion; CS, cardiac stomach; E, esophagus; EG, esophageal ganglion; IP, incisor part of mandible; lPC, lateral protocerebrum; MP, molar part of mandible; OL, olfactory lobe; PT, protocerebral tract; SS, cluster of sensory neurons; STG, stomatogastric ganglion; TeG, tegumental glands.

**FIGURE 8:**
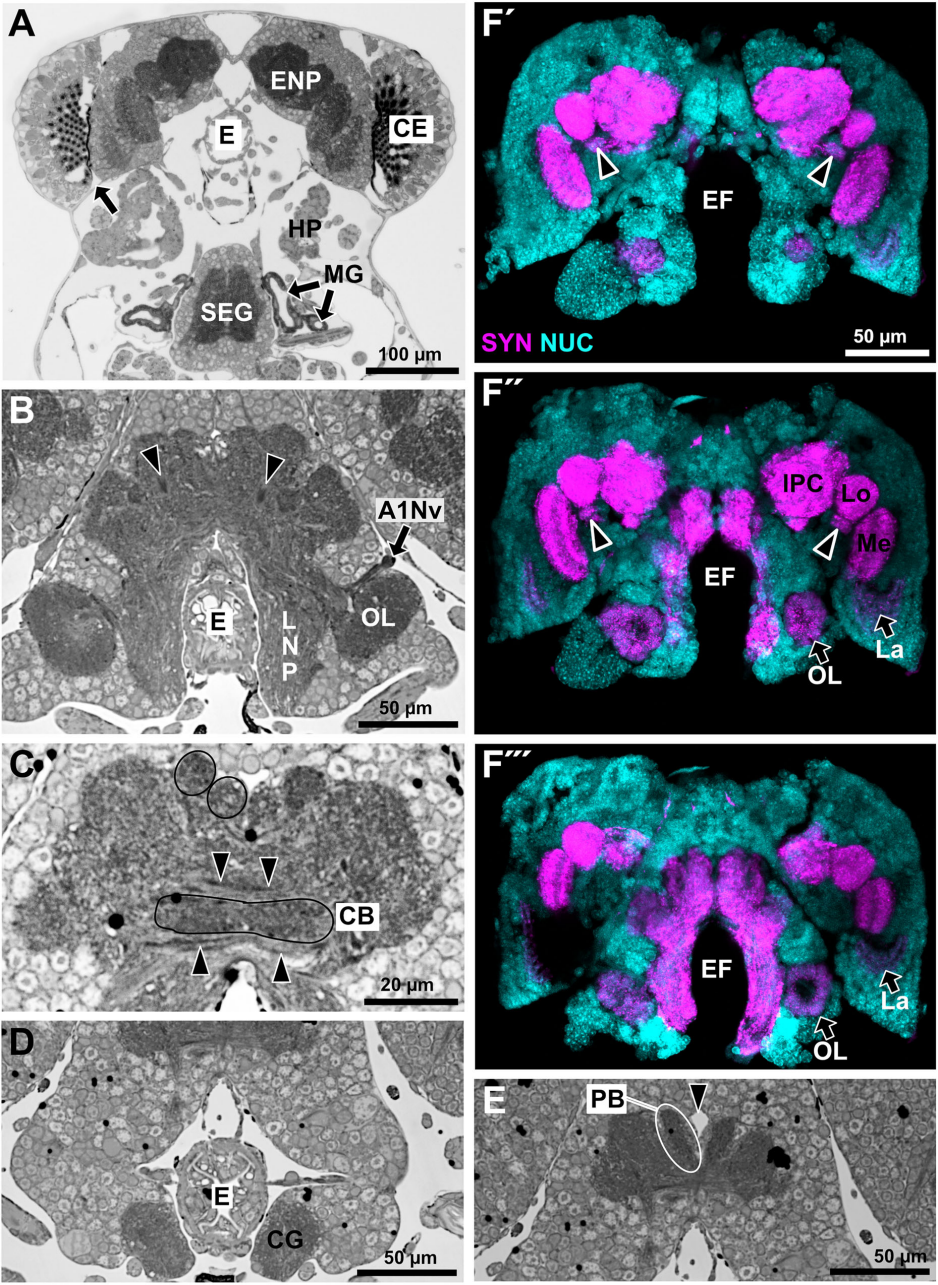
*Hemigrapsus sanguineus*, Zoea 1; (A–E) selected horizontal sections from a dorsal‐to‐ventral series (1.5 µm, stained with phenylene‐diamine) showing various details of the cephalic nervous system. The arrow in (A) identifies cuticular extensions that prefigure the posterior side of the developing eyestalks. Arrowheads in (B) identify the olfactory globular tract. Arrowheads in (C) identify commissural neurite bundles that extend in parallel to the central body. Circles in (C) identify spherical neuropils associated with the protocerebral bridge. The arrowhead in (E) identifies the cerebral artery. (F′–F‴) Dorsal‐to‐ventral series of single optical sections recorded with a confocal laser‐scan microscope (magenta: anti‐synapsin immunohistochemistry; blue: DNA marker to label nuclei). Arrowheads identify a satellite neuropil posteriorly to the lobula. A1Nv, nerve of antenna 1; CB, central body; CE, compound eye; CG, commissural ganglion; E, esophagus; EF, esophageal foramen; ENP, neuropils in the developing eyestalk; HP, hepatopancreas; La, lamina; Lo, lobula; lPC, lateral protocerebrum; Me, medulla; MG, maxillary gland; OL, olfactory lobe; PB, protocerebral bridge; SEG, subesophageal ganglia.

Within the developing, bilaterally paired eyestalks, a large spherical neuropil, the lateral protocerebum (lPC) takes a central position between the visual neuropils and the central brain (Figures 2C,D, 3A,B, 5A,B, and 8B). In adult *Hemigrapsus nudus*, this neuropil (formerly called “medulla terminalis”) is differentiated into a caudal and a rostral subdivision, the latter of which comprises the mushroom bodies (formerly called “hemiellipsoid bodies”; see Strausfeld and Sayre 2021). However, such differentiation is not yet visible in the larval lPC, but instead the neuropil appears to be rather homogenously structured. A short bundle of neurites, the protocerebral tract (PT), links the lateral protocerebrum to the median protocerebrum (Figure 7A). A dome‐shaped small neuropil is located dorsally to the lPC (double arrowheads in Figure 7A), perhaps an anlage of the later‐developing mushroom bodies (Strausfeld and Sayre 2021). The olfactory lobes (OLs) in the central brain are linked to the bilaterally paired lobes of the lateral protocerebrum via the olfactory globular tract (OGT). Profiles of this tract can be traced at various levels of the frontal and horizontal section series (single arrowheads in Figures 7A,B,D and 8B) so that a 3D reconstruction was possible using Amira (Figure 2, inset). The bilateral arms of the olfactory protocerebum cross over in the center of the central brain, near the central body (CB) (see below). A small, conspicuous bundle of neurites extends posteriorly from the lateral protocerebrum (single arrowheads in Figure 5A,A′, and inset) to target darkly stained tissue structures that border an extensive hemolymph sinus. Considering the location of this structure in reference to the studies by Webster and Dircksen (1991; their Figure 1), and Chung and Webster (2004, their Figure 2), who used immunohistochemistry against molt inhibiting hormone and crustacean hyperglycemic hormone, we tentatively identified this organ as the sinus gland (SG, Figure 5A,A′).

Within both developing eyestalks, we identified the developing Organ of Bellonci (OB) anteriorly to the lateral protocerebrum and the lobula. Specifically, we located one or several “onion bodies” (On), strongly stained aggregations of concentrically arranged membranous lamellae (Figure 6A–C). Because phenylenediamine was shown to have a high affinity for phospholipids (Shirai et al. 2016), we conclude that, consistent with ultrastructural studies on adult shore crabs *C. maenas* (Smith 1974), the larval onion bodies may be composed primarily of biomembranes. In both the horizontal (Figure 6A,A′), and frontal (Figure 6B,C,C′) section series, a characteristic cavity (Cv) with a diameter of around 20 µm is visible medially to the onion bodies, lined by a thin layer of membranous material. Furthermore, a small pore penetrates the cuticle anteriorly to the onion bodies providing access to the outside medium (arrowheads in Figure 6A,A′).

### 3.3 Central Brain and Stomatogastric Innervation

An elaborated complex of protocerebral neuropils, the longitudinal neuropils (LNPs), and the deutocerebral olfactory lobes (OLs) (Figure 2D,D′; and further details in the next section) characterizes the central brain. Remarkably, despite the various methods used here, we could not detect any neuropil representing the input of antennae 2 at this stage, suggesting that this representation develops during later larval life. Posteriorly, the commissural ganglia (CG; Figures 7F and 8D) flank the postesophageal commissure (PEC; Figure 2D′). Hemolymph is directed towards the central brain via the unpaired anterior aorta (single arrowheads in Figure 3A–D), which opens into a dorsal blood sinus (DS; Figure 3B) from where the unpaired cerebral artery descends ventrally across the brain (white arrowheads in Figure 3B,D). Because the eyestalks are not yet fully developed and not freely movable, this situation differs markedly from the blood supply in a Zoea 4 of *C. maenas* in which characteristic ophthalmic arteries supply the eyestalks and a characteristic myoarterial formation, the *core frontale*, amplifies the blood supply of the central brain (Spitzner et al. 2018).

The central brain is penetrated by the esophagus (E; Figures 2A, 7D, and 8A,B,D). The unpaired esophageal ganglion (EG) is anteriorly associated with the esophagus (Figure 7C). Furthermore, the unpaired stomatogastric ganglion (STG) could be identified slightly dorsal to the central brain (Figure 7A), anteriorly to the cardiac stomach (CS; Figure 7C).

**FIGURE 9:**
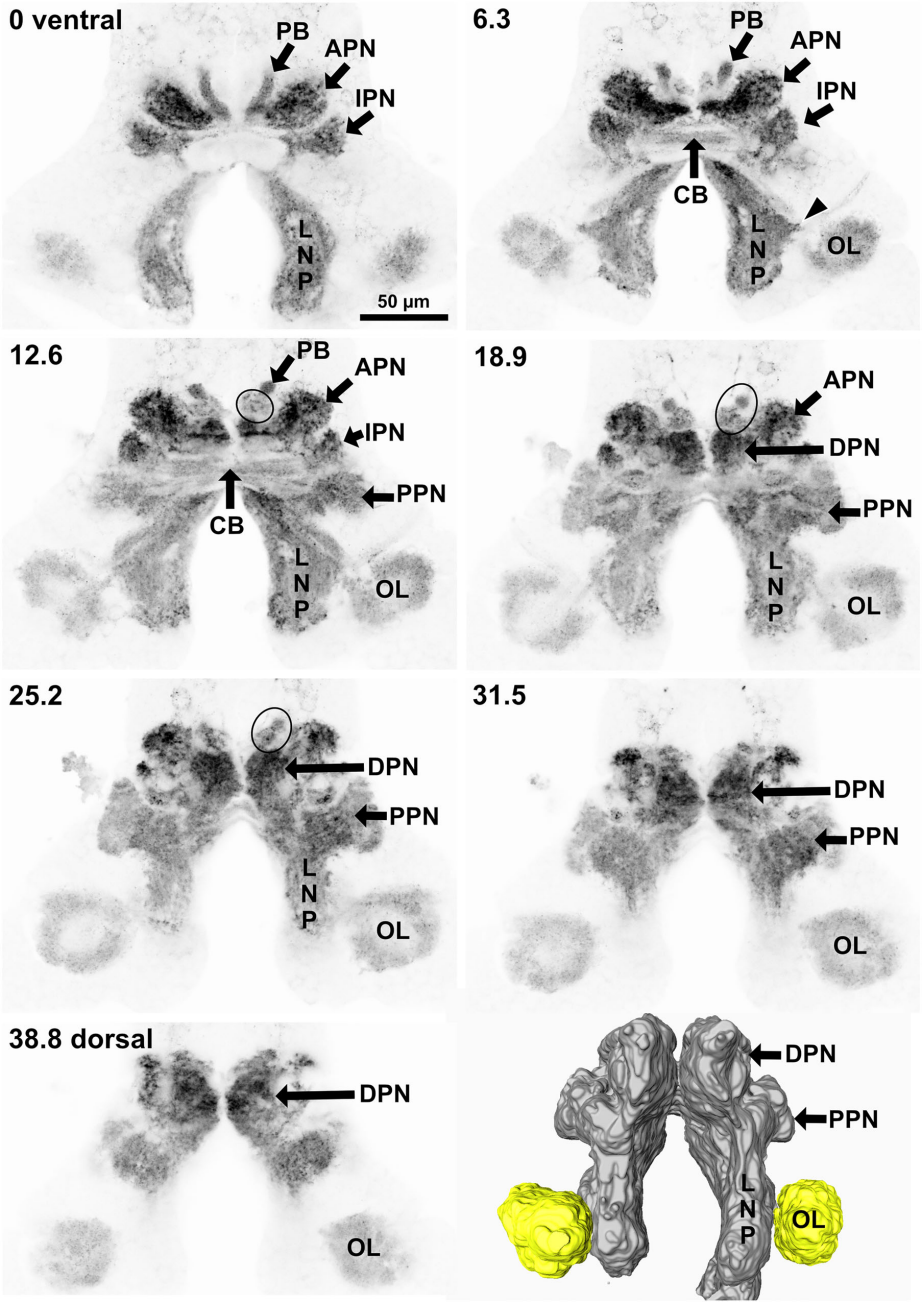
*Hemigrapsus sanguineus*, Zoea 1, central brain: ventral (“0”)—to—dorsal (“38.8”) series of single optical sections (black–white inverted images) recorded with a confocal laser‐scanning microscope, anti‐synapsin immunohistochemistry; numbers in the upper left corner (in µm) designate the *z*‐position of the optical sections within the image stacks with ventral defined as “0.” The image in the lower right represents a 3D reconstruction of this image stack (Amira). The scale bar applies to all images. APN, anterior protocerebral neuropil; CB, central body; DPN, dorsal protocerebral neuropil; IPN, intermediate protocerebral neuropil; LNP, longitudinal neuropil; OL, olfactory lobe; PB, protocerebral bridge; PPN, posterior protocerebral neuropil.

Immunohistochemistry against synapsins allowed for a fine‐grained analysis of the elements within the median protocerebrum (Figure 9). We were able to distinguish the bilaterally paired anterior protocerebral neuropils (APNs), intermediate protocerebral neuropils (IPNs), dorsal protocerebral neuropils (DPNs), and posterior protocerebral neuropils (PPN; Figure 9). An unpaired transverse, spindle‐shaped neuropil, the CB, is embedded within the protocerebral neuropils (Figures 7B, 8C, 9_12.6). It is anteriorly and posteriorly flanked by commissural neurite bundles (arrowheads in Figure 8C). In both horizontal histological sections (Figure 8E) and brains labeled with the anti‐synapsin antiserum (Figure 9_0_6.3_12.6), the two arms of the protocerebral bridge (PB), which is located anteriorly within the median protocerebrum, can be identified. It is associated with several less distinctly labeled, spherical neuropils (circles in Figures 8C and 9_12.6_18.9_25.2).

**FIGURE 10:**
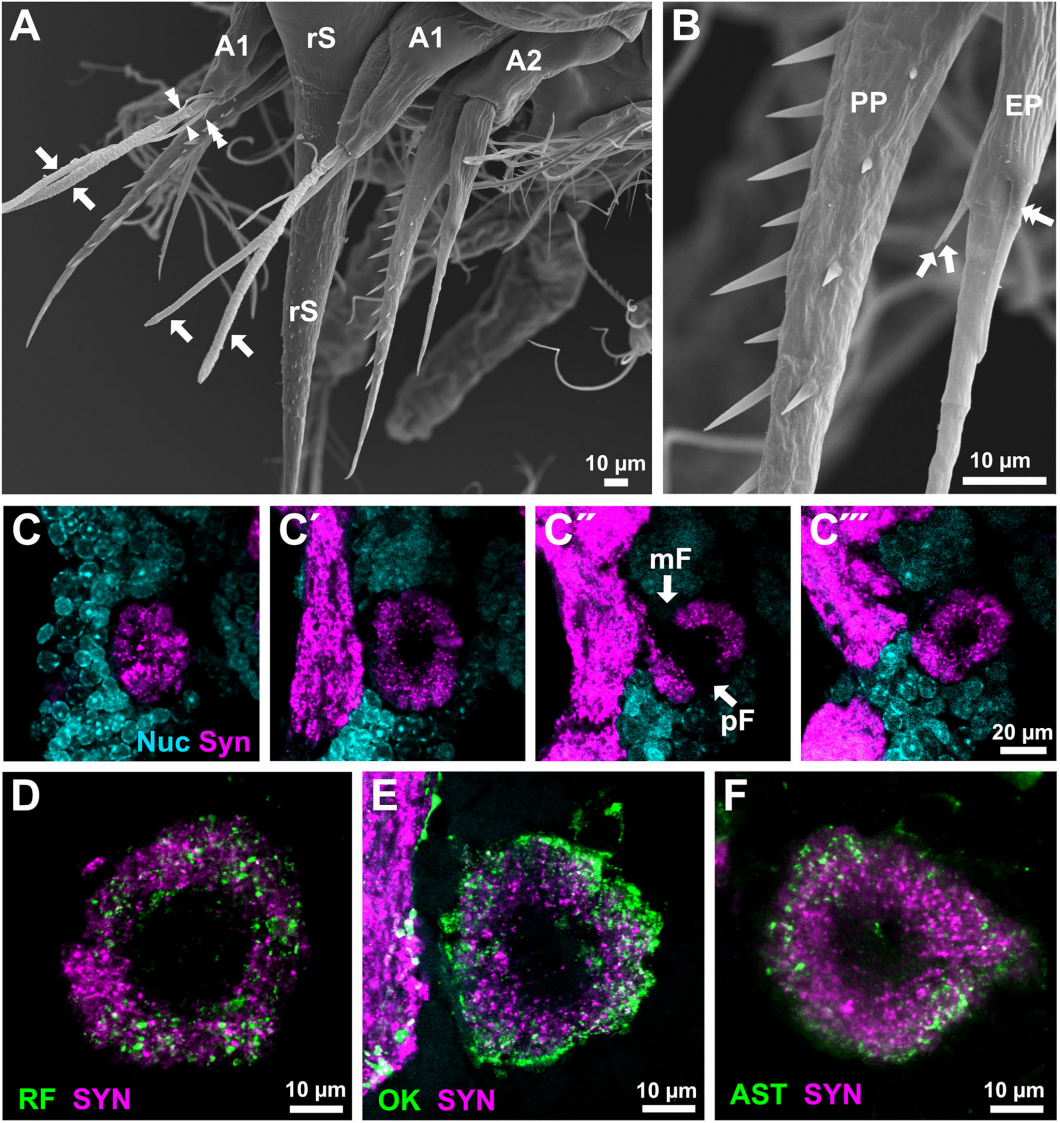
*Hemigrapsus sanguineus*, Zoea 1; (A and B) scanning electron micrographs showing the larval cephalic sensory appendages. Arrows in (A) identify the long and slender aesthetascs, the single arrowhead a shorter aesthetasc, and the double arrowheads one longer and triple arrowheads one shorter simple seta on the first of antennae. In (B), the denticles of the elongated spinous process (protopod) of antenna 2 are visible. Single arrows identify two smaller setae, and the double arrow one larger seta at the tip of the exopod. (C–C‴) olfactory lobes labeled by anti‐synapsin immunoreactivity (magenta) and nuclear counterstain (blue); dorsal (C) to ventral (C‴) series of single optical sections (ca. 10 µm *z*‐distance between the optical sections). (D–F) Olfactory lobes double‐labeled against neuropeptides (green) and synapsins (magenta) showing RFamide‐like immunoreactivity (D), orcokinin‐like immunoreactivity (E), and allatostatin‐like immunoreactivity (F). A1, antenna 1; A2, antenna 2; EP, exopod of antenna 2; mF, median foramen; pF, posterior foramen; PP, protopod of antenna 2; rS, rostral spine.

The central brain also comprises the OLs that will be described in the next section. The bilaterally paired LNPs are unstructured blocks of neuropil that posteriorly adjoin the various protocerebral neuropils and extend posteriorly, on both sides flanking the esophageal foramen (Figures 8B and 9). Posteriorly, the LNPs are adjoined by the CG (Figure 2D,D′). Our approach revealed only a few substructures within these neuropils. A lateral extension (arrowhead in Figure 9_6.3) may represent a first anlage of the antenna 1 neuropil. Remarkably, although the tritocerebral pair of antennae is well developed and features an elongated spinous process (protopod) with numerous denticles and an exopod with three terminal simple sensilla (Figure 10A,B; compare also Lee and Ko 2008), within the LNPs—using our methodological repertoire—we could not identify any substructures representing an input from, or output to, the second antennal pair.

### 3.4 The Olfactory Pathway

Matching the description of Lee and Ko (2008), we found the uniramous first pair of antennae (“antennulae”) being equipped with two long, slender aesthetascs (arrows in Figure 10A), one shorter, thinner aesthetasc (arrowhead), and one longer (double arrowheads) and one shorter (triple arrowheads) simple seta (Figure 10A). The somata of the sensory neurons associated with these sensilla are arranged in a bilaterally paired compact cluster located just ventral to the brain and extending into the proximal part of the first antennae where this appendage is attached to the cephalothorax (SS in Figures 2D′, 3B, 7B). From these clusters of sensory neurons, the bilaterally paired antenna 1 nerve (A1Nv, Figures 7C and 8B) extends towards the brain to innervate the OLs, conspicuous spherical neuropils within the deutocerebrum (Figures 2D,D′, 7D, 8B,F″,F‴, 9). Our analysis of individual optical sections of brain wholemounts together with the immunolocalization of synapsins revealed that the synaptic areas are confined to a cortical layer of the lobe, whereas the central core does not contain any synaptic material (Figure 10C–C‴). Therefore, in central optical sections across the lobe, the synaptic layer has the shape of a doughnut (Figure 10C′,D–F). The synaptic layer has two characteristic unlabeled gaps, the median (mF) and posterior (pF) foramina, through which, presumably, olfactory interneuron neurites enter and exit the lobes. Immunohistochemistry against neuropeptides revealed a diverse neurochemistry of the lobe's synaptic neuropil (Figure 10D–F). Orcokinin‐like and allatostatin‐like immunoreactive material is located more towards the periphery of the lobe, whereas RFamide‐like immunoreactive material appears to be evenly distributed across the synaptic layer of the lobe. The OGT exits the OLs in a medial direction (arrowheads in Figure 7D), and profiles of this tract can be traced in various levels of the frontal and horizontal section series as it approaches the lateral protocerebrum (single arrowheads in Figures 7A,D and 8B). A 3D reconstruction (Figure 2, inset) showed that its bilaterally arranged arms form a chiasm in the center of the central brain near the CB.

### 3.5 Second Antennae

Matching the description of Lee and Ko (2008), the long spinous process (protopod) of the biramous second antennae is decorated with two parallel rows of pointed denticles (*sensu* Garm 2004; Figure 10B). The endopod bears two smaller setae (arrows in Figure 10B), and one larger seta at the tip (double arrow in Figure 10B) indicating a sensory function. For the spinous process, its armature with denticles suggests a defensive function (perhaps in cooperation with the rostral spine) or a function in the context of handling food, or in grooming the sensilla on the first antennae (cf., Garm and Watling 2013).

### 3.6 Neurochemistry of the Brain

**FIGURE 11:**
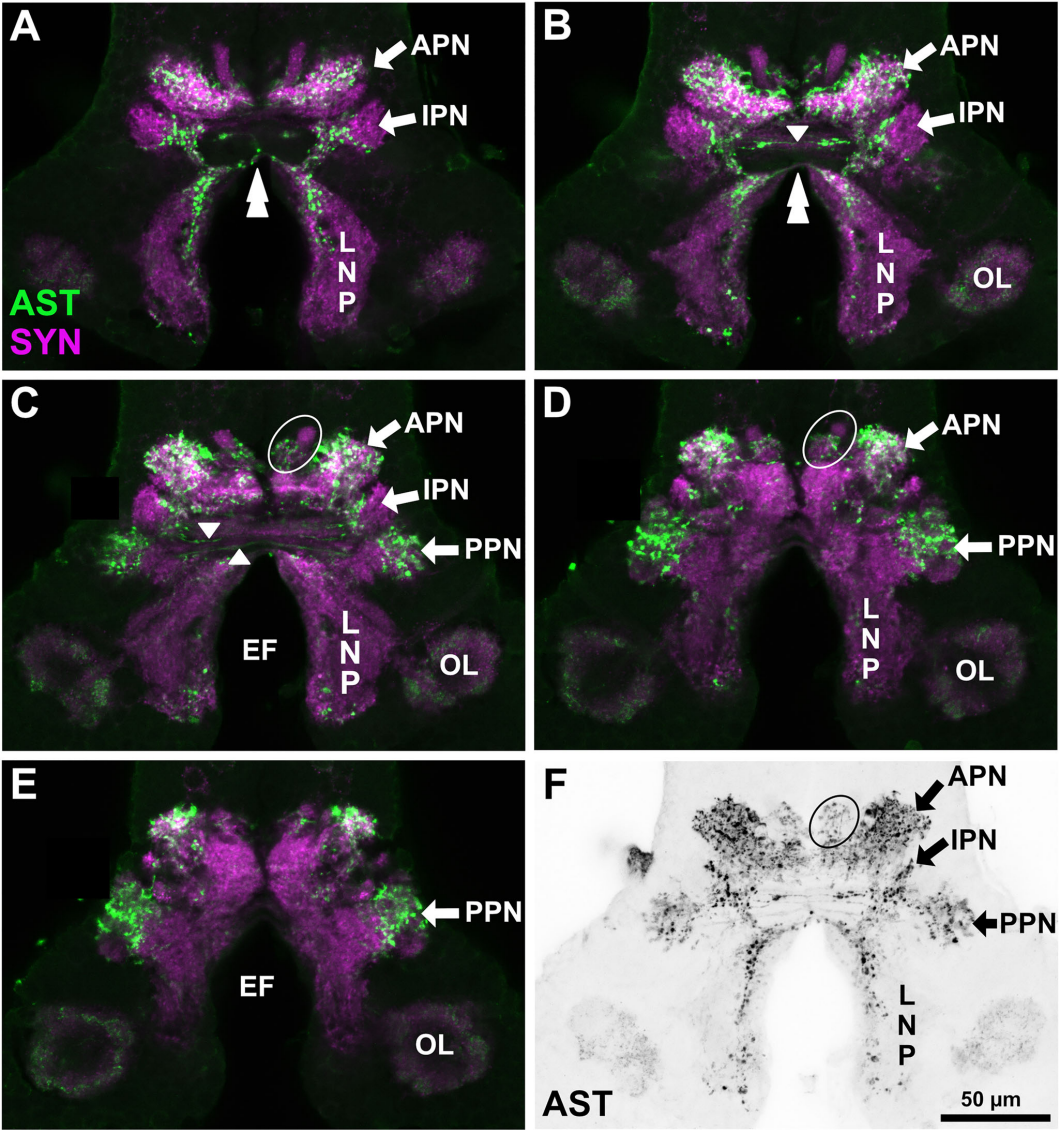
*Hemigrapsus sanguineus*, Zoea 1: immunohistochemistry against allatostatin‐like neuropeptides (AST, green) and synaptic proteins (SYN, magenta), confocal laser‐scan microscopy. (A–E) Each image is a *z*‐projection of five optical planes of 0.63 µm thickness from one representative central brain (A ventral, E dorsal). The distance between the individual section planes is ca. 3 µm. The double arrowheads in (A) and (B) identify commissural neurites just anterior to the esophageal foramen. Single arrowheads in (B) and (C) identify commissural neurites associated with the central body. Circles in (C and D) and (F) identify a spherical neuropil associated with the protocerebral bridge. (F) AST channel, *z*‐projection of 10 optical planes of 0.63 µm thickness, black–white inverted image. Scale bar in F relates to all images in this panel. APN, anterior protocerebral neuropil; EF, esophageal foramen; IPN, intermediate protocerebral neuropil; LNP, longitudinal neuropil; OL, olfactory lobe; PPN, posterior protocerebral neuropil.

Strong allatostatin‐like immunoreactivity (ASTir) was detected in the APNs and PPNs, and slightly less intense, also in the IPN (Figure 11A–F). However, we failed to trace any somata showing ASTir. A small spherical neuropil associated with the PB showed weak ASTir (circles in Figure 11C,D,F). A conspicuous transverse neurite showing ASTir is embedded within the CB (arrow head in Figure 11B; and see 11F). Additional commissural neurites accompany the CB posteriorly (single arrowheads in Figure 11B,C). Commissural neurites displaying ASTir link the hemibrains just anteriorly to the esophageal foramen (double arrowheads in Figure 11A,B). The LNP displays diffuse ASTir, albeit less intense than that in the protocerebral neuropils. Furthermore, compared with the aforementioned structures, ASTir in the OLs is much less intense (Figure 11F) and can be visualized only with higher gain (Figure 10F).

**FIGURE 12:**
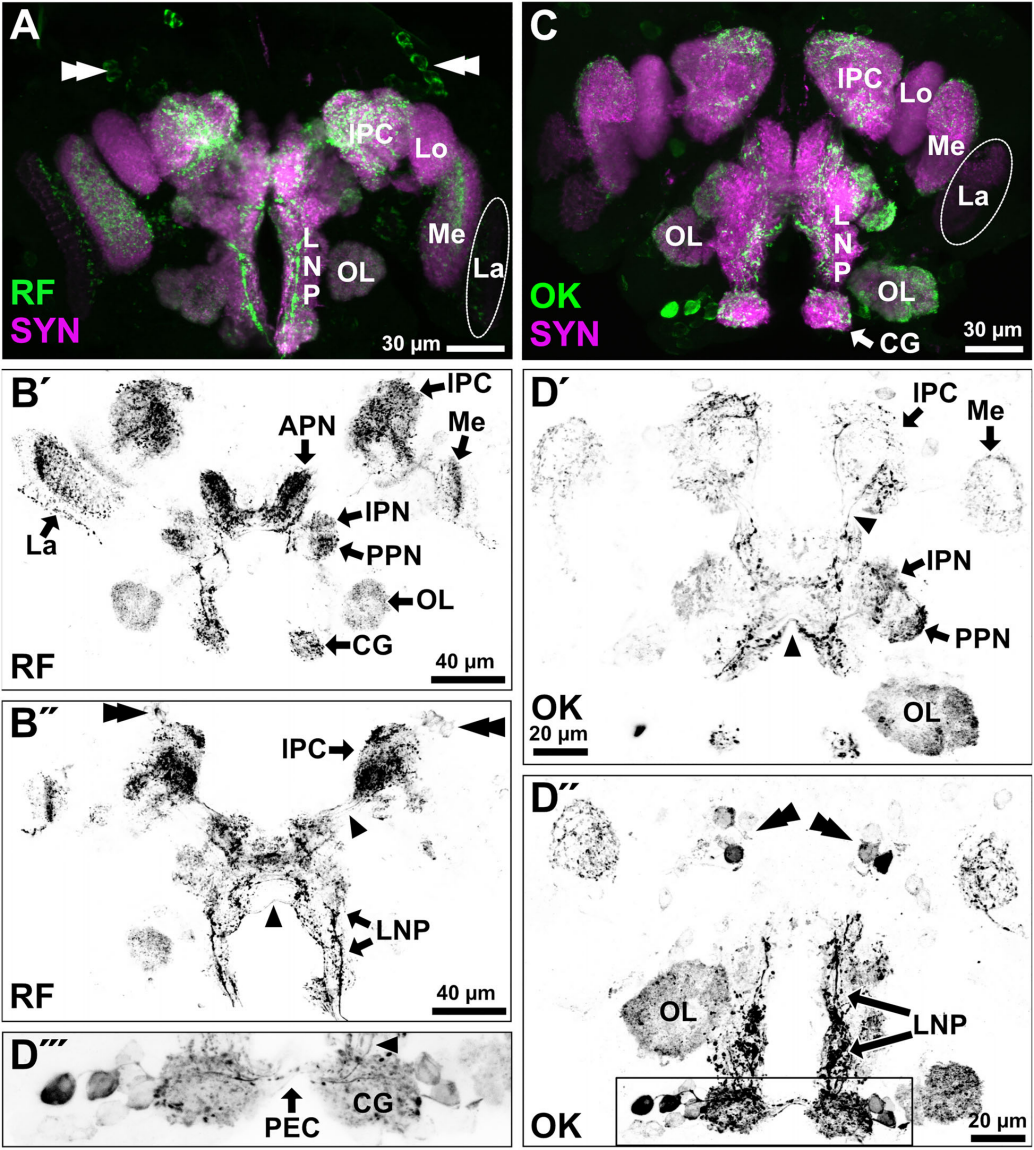
*Hemigrapsus sanguineus*, Zoea 1; (A, B′, B″) immunohistochemistry against RFamide‐like neuropeptides (RF, green) and synaptic proteins (SYN, magenta), confocal laser‐scan microscopy. B′ and B″ display the RFamide signal only (black–white inverted images). Each image is a *z*‐projection of five optical planes of 0.63 µm thickness from one representative central brain. The double arrowheads in (A) and (B″) identify a cluster of neuronal somata innervating the lateral protocerebrum. The single median arrowhead in (B″) identifies a commissural neurite at the posterior margin of the central brain, and the arrowhead in the right half of the panel identifies a neurite within the protocerebral tract linking the lateral protocerebrum and the central brain. (C, D′–D‴) Immunohistochemistry against Orcokinin‐like neuropeptides (OK, green) and synaptic proteins (SYN, magenta), confocal laser‐scan microscopy. (D′–D‴) Display the Orcokinin signal only (black–white inverted images). Each image is a *z*‐projection of five optical planes of 0.63 µm thickness from one representative central brain. The single median arrowhead in (D′) identifies commissural neurites at the posterior margin of the central brain, and the arrowhead in the right half of the panel neurites within the protocerebral tract linking the lateral protocerebrum and the central brain. The double arrowheads in (D″) identify a cluster of neuronal somata innervating the lateral protocerebrum. (D‴) is a higher magnification of the boxed area in (D″) showing strongly labeled neurons with neurites that cross to the contralateral side in the postesophageal commissure to then veer anteriorly (arrowhead). APN, anterior protocerebral neuropil; CG, commissural ganglion; IPN, intermediate protocerebral neuropil; La, lamina; LNP, longitudinal neuropil; Lo, lobula; lPC, lateral protocerebrum; Me, medulla; OL, olfactory lobe; PEC, postesophageal commissure; PPN, posterior protocerebral neuropil.

RFamide‐like immunoreactivity (RFir) is present in the somata of six to eight large neurons (double arrowheads in Figure 12A,B″) in both developing eyestalks, the neurites of which strongly innervate the lateral protocerebrum (Figure 12A,B′,B″). While the lobula seems devoid of labeling, RFir is present in the lamina and medulla. Immunolabeled fine neurites within the PT (arrowhead in Figure 12B″) extend from the lateral protocerebrum towards the central brain. Within the median protocerebrum, the anterior, intermediate, and PPNs display distinct RFir (Figure 12B′). The LNPs also display distinct immunoreactive profiles, as do the CG. Fine commissural neurites (median arrowhead in Figure 12B″) link the bilaterally arranged LNPs. As described for ASTir in the previous section, RFir in the OLs (Figures 10D, 12B′) is relatively weak when compared with the protocerebral neuropils.

Orcokinin‐like immunoreactivity (OKir) is present in the medulla and lateral protocerebrum (Figure 12C,D′) and originates from two strongly stained, bilaterally paired somata (double arrowheads in Figure 12D″) located ventrally within the developing eyestalks. The distal portions of the intermediate and PPNs are also strongly immunoreactive. As with the two previously mentioned neuropeptides, commissural neurites displaying OKir at the posterior margin of the central brain (medial arrowhead in Figure 12D′) link the two hemibrains. OKir is particularly strong in the LNPs, where numerous coarse neurites arranged in a longitudinal direction are visible. This innervation likely originates from bilaterally paired cell clusters that comprise two strongly OKir somata and several weakly labeled ones (boxed area in Figure 12D″,D‴). The neurites of the strongly labeled somata most likely provide an innervation of the CG, then cross over to the contralateral side in the PEC (Figure 12D‴) where they extend further anteriorly (arrowhead in Figure 12D‴) towards the LNP (Figure 12D″). OKir within the OLs has a fine granular texture (Figures 10E and 12D′,D″).

**FIGURE 13:**
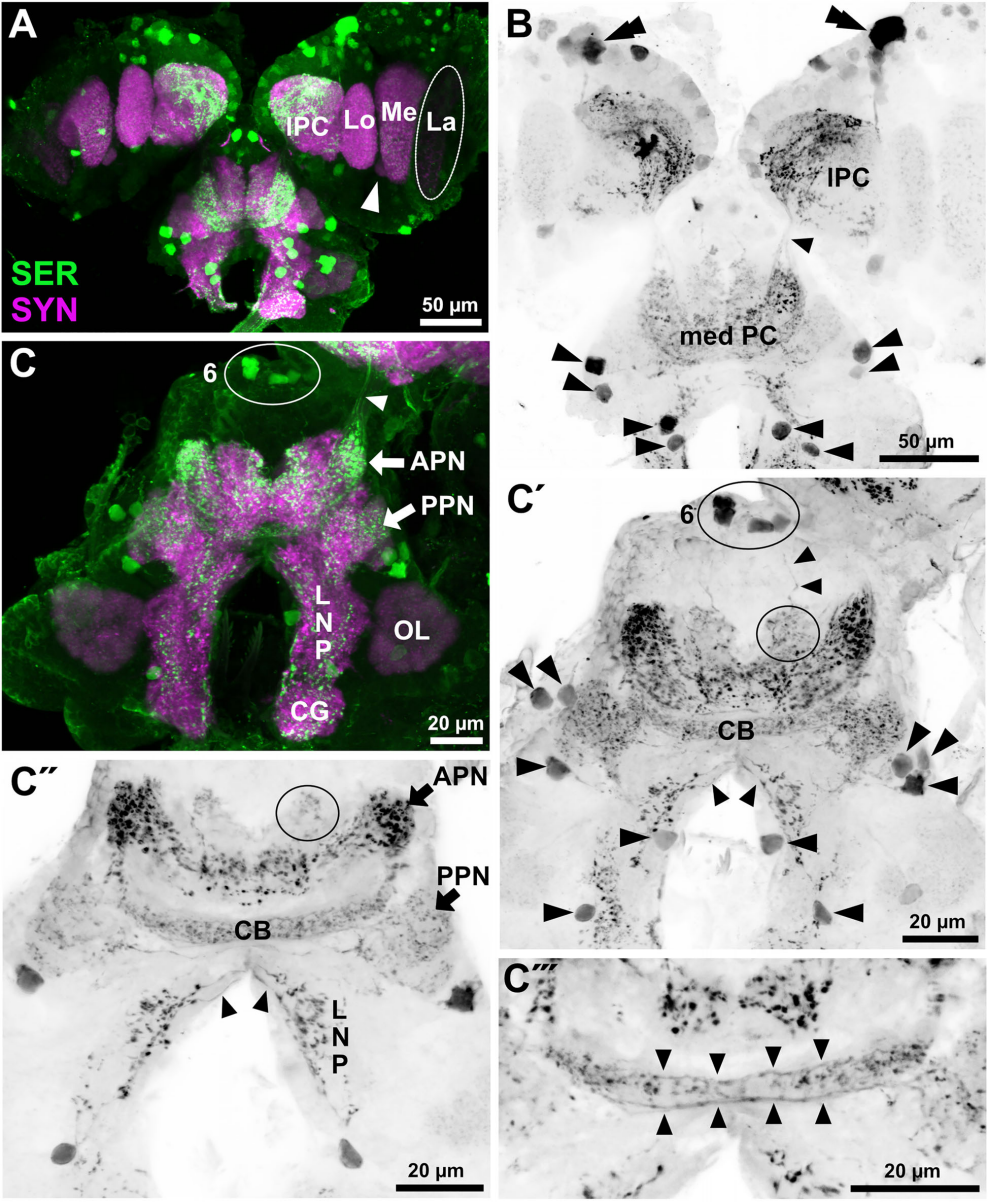
*Hemigrapsus sanguineus*, Zoea 1: immunohistochemistry against serotonin (SER, green) and synaptic proteins (SYN, magenta), confocal laser‐scan microscopy. Parts (B–C‴) display the serotonin signal only (black–white inverted images). Part (A) is a *z*‐projection of five optical planes of 0.63 µm thickness from one representative central brain; (B and C‴) represent single optical sections. The arrowhead in (A) identifies the visual satellite neuropil. The double arrowheads in (B) identify clusters of neuronal somata innervating the lateral protocerebrum. The single arrowheads on the right sides of (B) and (C) identify neurites extending from the lateral protocerebrum to the median protocerebrum. The left‐right symmetrically arranged arrowheads in (B) and (C′) label neuronal somata. In (C′), somata in cell cluster (6) (oval) extend neurites (arrowheads) towards a spherical neuropil (circle). Medially arranged arrowheads in (C′) and (C″) identify commissural neurites at the posterior margin of the central brain. Arrowheads in (C‴) identify transverse neurites that accompany the central body anteriorly and posteriorly. 6, cell cluster (6); APN, anterior protocerebral neuropil; CB, central body; CG, commissural ganglion; La, lamina; LNP, longitudinal neuropil; Lo, lobula; lPC, lateral protocerebrum; Me, medulla; med PC, median protocerebrum; OL, olfactory lobe; PPN, posterior protocerebral neuropil.

Strong serotonin‐immunoreactivity (SERir) is present in the lateral protocerebrum (Figure 13A,B) and originates from bilaterally paired clusters of strongly stained somata (double arrowheads in Figure 13B). Numerous small, weakly labeled SERir somata are scattered throughout the cell cortex of the developing eyestalks (Figure 13A). Within the neuropil in the lamina, medulla, and lobula, a staining that is only barely detectable above background is present (Figure 13B). Fine neurites with SERir link the lateral protocerebrum to the median protocerebrum (arrowhead in Figure 13B,C). There, the APNs and PPNs display strong staining (Figure 13C,C″). Weak SERir is present in the spherical neuropils (circle in Figure 13C′), which are associated with the PB. Immunolabeled somata in the anteriorly located cell cluster (6; Figure 13C,C′) extend weakly labeled neurites into this brain region (anteriorly located arrowheads in Figure 13C′). The CB is also clearly labeled (Figure 13C′,C″), and distinct transverse neurites accompany its neuropil core anteriorly and posteriorly (arrowheads in Figure 13C‴). Strongly labeled single, and pairs of SERir somata are arranged in a left/right symmetrical pattern within the central brain (pairs of arrowheads in Figure 13B, single arrowheads in the lower half of Figure 13C′). The LNPs and CG also display distinct immunoreactive profiles (Figure 13C,C″), and fine commissural neurites at the posterior side of the central brain (median arrowheads in Figure 13C′,C″) link these bilaterally arranged neuropils. The OLs appear devoid of any SERir signal.

### 3.7 Ventral Nerve Cord and Peripheral Release Sites

**FIGURE 14:**
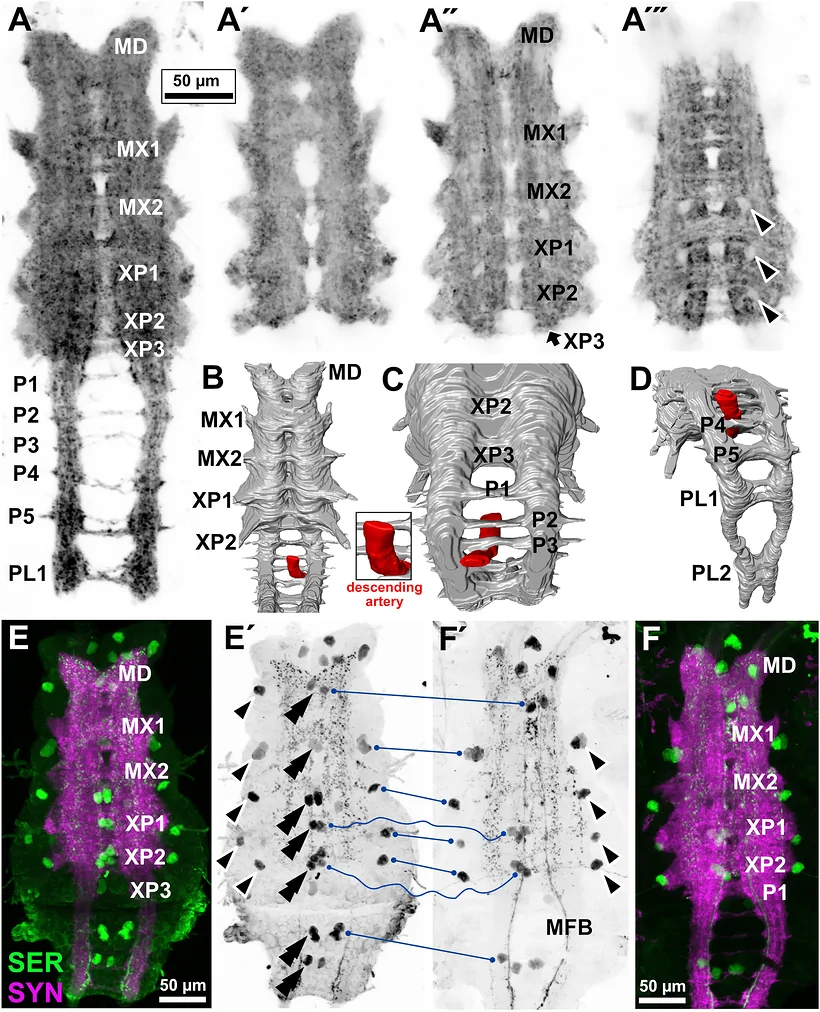
*Hemigrapsus sanguineus*, Zoea 1. (A–A‴) Selected optical sections (confocal laser‐scan microscopy) of a ventral nerve cord (whole mount), labeled for synaptic proteins to identify neuropil regions (black/white inverted image). The scale bar in (A) applies to all individual images. The arrowheads in (A‴) identify patches of unlabeled material that are serially arranged and embedded within synaptic neuropil. (B–D) three‐dimensional reconstruction (Amira) of the ventral nerve cord generated from a frontal section series (136 sections, 1.5 µm thickness, stained with phenylene‐diamine). Part (B) shows a ventral aspect, (C) a dorsal view, and (D) a caudal view. (E and F) Immunohistochemistry against serotonin (SER, green) and synaptic proteins (SYN, magenta), confocal laser‐scan microscopy. Parts (E′, F′) display the serotonin signal only (black–white inverted images). Two different specimens are shown. Single arrowheads in (E′) and (F′) identify laterally located somata, and double arrowheads medially located somata. Blue lines connect corresponding single or pairs of immunolabeled somata that are present in both specimens. MD, neuromere of the mandible; MFB, medial fiber bundle; MX1/2, neuromeres of maxilla 1/2; P1–5, neuromeres of the later‐developing pereiopods 1–5; PL1/2, pleon ganglia 1/2; XP1/2/3, neuromeres of maxilliped 1–3.

As revealed by anti‐synapsin immunohistochemistry (Figure 14A–A‴) and 3D reconstruction of serial histological sections (Figure 14B–D), in the developing VNC, the neuromeres that are associated with functional appendages are well developed: neuromeres of the mandible (MD), maxillae 1 and 2 (MX1, 2), maxilliped 1 and 2 (XP1, 2). Although these neuromeres show a high degree of fusion, transverse commissural fibers and nerve roots extending towards the periphery indicate their underlying segmental organization. In the immunohistochemical preparations, unidentified patches of unlabeled material are serially arranged and embedded within synaptic neuropil (arrowheads in Figure 14A‴). Posteriorly, the developing neuromere associated with the segment of maxilliped 3 (XP3) is followed by anlagen of the ganglia associated with the segments of the (later developing) pereiopods 1–5 (P1–P5). The descending artery penetrates the VNC between the neuromeres P3 and P4. Further posteriorly, the rudimentary ganglia of pleomeres 1 and 2 follow, transversely linked by segmental commissures (PL1, 2; Figure 14A–D). We were unable to reconstruct the more caudal pleon ganglia because these were cut off during dissection.

Anti‐serotonin immunoreactivity (SERir) showed a high level of variability in labeling intensity between individual specimens, a phenomenon that may reflect the animal's physiological state prior to fixation and fluctuations of serotonin levels during the process of fixation (Stemme and Stern 2015). Therefore, we decided to show two different specimens that we consider representative to illustrate this variability (see Figure 14E,E′,F,F′). Two types of SERir neurons are present in the developing nerve cord, one type with laterally arranged somata (single arrows in Figure 14E′,F′), and a second type with medially arranged somata (double arrowheads in Figure 14E′,F′). All neuromeres down to the maxilliped 2 segment contain somata of both types. Most of the SERir somata are arranged in a bilaterally symmetrical pattern, and some somata could be identified in both specimens presented here (blue lines in Figure 14E′,F′). A bundle of medially arranged neurites (MFB) extended across the length of the VNC. However, the quality of our preparations was not sufficient to analyze the morphology of neurites associated with the individual neuron types as we previously did in the VNC of embryonic American lobsters *H. americanus* (Harzsch 2003a).

**FIGURE 15:**
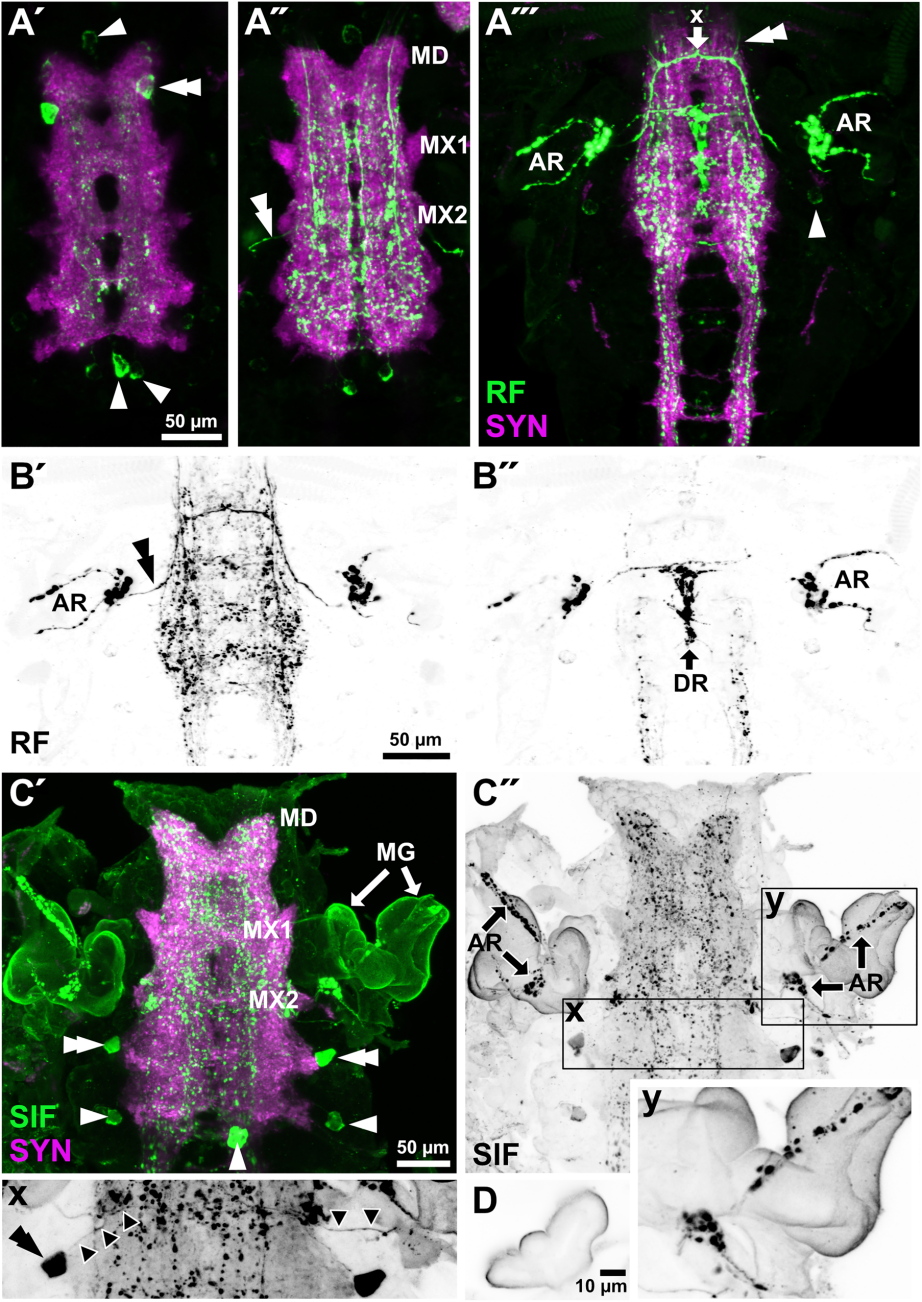
*Hemigrapsus sanguineus*, Zoea 1; (A–A‴), (B′ and B″) immunohistochemistry against RFamide‐like neuropeptides (RF, green) and synaptic proteins (SYN, magenta), confocal laser‐scan microscopy (A′ is a composite of 5 optical sections, A″ of 10 optical sections, and A‴ of 60 optical sections. B′ is a composite of 20 optical sections, B″ of 25 optical sections). Parts (B′) and (B″) display the RFamide signal only (black–white inverted images). Single arrowheads in (A′) and (A‴) identify immunolabeled somata. The double arrowhead in (A′) labels a conspicuous soma, the neurite of which crosses to the contralateral side (X in A‴) to exit the ventral nerve cord (double arrowhead in A″ and B′) and spread out in the periphery to form the anterior ramifications (A‴) and the dorsal ramifications (B″). (C′, C″, and D), **and insets**: immunohistochemistry against SIFamide‐like neuropeptides (SIF, green) and synaptic proteins (SYN, magenta), confocal laser‐scan microscopy (C′ is a composite of 95 optical sections, and (C″) of 130 optical sections. (D) is a composite of 15 optical sections). Parts (C″, D) and insets display the SIFamide signal only (black–white inverted images). The inset labeled X is a higher magnification of the boxed area in (C″), and the inset labeled Y a higher magnification of the second boxed area in (C″). Single arrowheads in (C′) identify immunolabeled somata. Double arrowheads in (C′) identify bilaterally paired somata, the neurites of which cross to the contralateral side (arrowheads in the boxed area with a single dot) to exit the ventral nerve cord and expand within the anterior ramifications (boxed area with two dots). Part (D) is a higher magnification of the maxillary gland. AR, anterior ramifications; DR, dorsal ramifications; MD, neuromere of the mandible; MG, maxillary gland; MX1/2, neuromeres of maxilla 1/2.

RFamide‐like immunoreactivity (RFir) is present in the somata of several neurons (single arrowheads in Figure 15A′,A‴) associated with the developing VNC, as well as the neuropils of the neuromeres of mandible, maxillae 1, and 2. A conspicuously large and bilaterally paired soma displaying RFir (double arrowhead in Figure 15A′) extends a neurite that crosses to the contralateral side (X in Figure 15A‴) to exit the VNC (double arrowheads in Figure 15A″,B′) and spread out in the periphery to form the strongly immunoreactive anterior ramifications (AR; Figure 15A‴,B′). These anterior ramifications presumably represent a developing neurohemal release site (see Pulver and Marder 2002). In newly hatched larvae of *C. maenas*, another neurohemal release site is present, the pericardial organs (Chung and Webster 2004), but unfortunately, we failed to label this organ in *H. sanguineus*. The same pair of cells also innervates a medially located plexus of strongly immunolabeled material located at the dorsal margin of the VNC that we named “dorsal ramifications” (DR; Figure 15B″).

SIFamide‐like immunoreactivity (SIFir) is present in the somata of several neurons (single arrowheads in Figure 15C′) that give rise to a network of SIFir within the neuropil of the neuromeres of the mandible, maxillae 1 and 2. Bilaterally paired somata (double arrowheads in Figure 15C′) extend neurites which cross to the contralateral side (arrowheads in the boxed area X) to exit the VNC and extend towards the strongly stained anterior ramifications (AR in Figure 15C″; and the boxed area Y). The anterior ramifications are closely associated with the bilaterally paired MG (Figure 15C′,C″). This tissue displays a diffuse signal of SIFir (Figure 15D) that we nevertheless believe to represent specific staining.

## 4 Discussion

### 4.1 Visual System

The principal morphology of the ommatidia in the Zoea 1 of *H. sanguineus* strongly resembles that of other larval Brachyura such as *C. maenas* and *Hyas araneus* (Harzsch and Dawirs 1995/96). The eyes are sessile in the Zoea 1 of *H. sanguineus* but will become stalked and mobile in the Zoea 2. It is well known that placing a CE on a stalk, as is typical for crabs, provides an expanded visual field, and the potential for mobility of the eye as the animal or its visual targets move (reviewed in Cronin and Porter 2008). The larval eyes of most decapod crustaceans, including brachyuran crabs, function according to the apposition optic principle (Nilsson 1983; Nilsson et al. 1986, reviewed in Cronin and Porter 2008). The adult eye of *H. sanguineus* continues to function as an apposition eye. Its morphology and circadian pigment migration were described by Arikawa et al. (1987). Generally, in Brachyura, the apposition optics persists throughout development (reviewed in Cronin and Porter 2008), but the number of ommatidia within the CEs increases substantially. We found only around 110 ommatidia per larval eye, whereas juvenile *H. sanguineus* of 5 mm carapace width possess around 2,000, and adults up to 6,000 (ca. 30 mm carapace width; Eguchi et al. 1989). In another representative of the taxon Varunidae, *Chasmagnatus/Neohelice granulata*, the adult CE was reported to comprise ca. 7,000 to 9,000 ommatidia (Sztarker et al. 2005; Tomsic et al. 2017). Considering that each ommatidium's rhabdom houses eight photoreceptor cells (reviews, e.g., Stavenga and Hardie 1998; Warrant and Nilsson 2006; Glantz 2014), postembryonically, the photoreceptor input to the central visual pathway in *H. sanguineus* increases by a factor of almost 50, from ca. 900 photoreceptor cells to ca. 46,000 in adults. This process must coincide with a dramatic change in the eye's interommatidial angle, which is a measure of the eye's resolution (Stavenga and Hardie 1998; Warrant and Nilsson 2006). We estimate the interommatidial angle of the *H. sanguineus* Zoea 1 to be close to that of *H. araneus* and *C. maenas* larvae, between ca. 9° and 13° (Harzsch and Dawirs 1995/96). This angle decreases to ca. 2° in adult *H. sanguineus* (Eguchi et al. 1989).

Crustacean eyes grow by the addition of new elements to the ommatidial array from the side (Harzsch et al. 1999; Melzer et al. 2000; Harzsch and Waloszek 2001; review: Harzsch and Hafner 2002). This growth is driven by a proliferation zone along the side of the eye that houses mitotically active cells as revealed by an s‐phase specific proliferation marker (Harzsch and Dawirs 1995/96; Harzsch et al. 1999). Although we did not apply any specific mitosis marker in the present study, our histological sections revealed a band of undifferentiated cells along the margin of the larval eyes that resembles the proliferation zone in *C. maenas* and *H. araneus* larvae (Harzsch and Dawirs 1995/96). These authors also showed sections across the eye center (their Figure 4A,B) in which ommatidia were visible in a tangential aspect and arranged in a radial array, and in the same section an additional field of ommatidia, which were 90° tilted and visible in a transverse aspect much like the arrangement that we report here for *H. sanguineus*. These findings—not recognized by Harzsch and Dawirs (1995/96)!—collectively indicate that in early zoeal stages of brachyuran larvae, the ommatidia may not be arranged in a perfect radial array as in the adults but are packed in a different pattern, a phenomenon that may be related to the fact that the eyes are not yet stalked.

The dominant visual neuropils in the *H. sanguineus* Zoea 1 suggest a remarkable investment of this organism into neuronal capacity for visual processing, paralleling the situation in the zoeal stages of other decapod crustaceans (Rotllant et al. 1995; Harzsch and Dawirs 1995/96; Geiselbrecht and Melzer 2013; Cronin et al. 2017). In addition to the well‐developed lamina, medulla, and lobula, we observed, in both eyestalks, a small SN located posteriorly between the tips of the medulla and lobula. Similarly, such SNs were localized in late embryos of the American lobster *H. americanus* (Harzsch 2002; his Figure 1B,C) and the Zoea 1 of the caridean shrimp *Hippolyte inermis* and the anomuran crab *Porcellana platycheles* (Geiselbrecht und Melzer 2013; their Figure 2). The location of these neuropils makes it impossible to determine if they derive from, or are associated with, either the medulla or lobula. To determine if this SN is indeed the anlage of the lobula plate that characterizes the visual system of, for example, adult *H. oregonensis* and *C./N. granulata* (Sztarker et al. 2009; their Figure 1; Barnatan et al. 2022) and other crustaceans (discussed in Strausfeld 2021) requires additional studies of larval and juvenile stages using techniques to visualize the fiber connections of the SNs (e.g., Sztarker et al. 2009).

### 4.2 The Organ of Bellonci/Sensory Pore X‐Organ

Anger (2001) has provided an overview of previous studies on the larval OB in malacostracan crustaceans. In the larval eyestalks of various species of *Palaemonetes*, Hubschman (1963), using the older name “sensory pore X‐organ” (SPX), localized the organ in a similar position as we did, close to the neuropil of the lPC (previously called terminal medulla). He described a cuticular pore (“sensory pore”), a vacuole, and onion bodies, named after the concentrical appearance of the membranous lamellae, as essential elements of the larval SPX (Hubschman 1963; their Figure 15). This author also described the organ's subsequent ontogeny during larval development, which is characterized by an increasing number and size of onion bodies and by droplets, that stain with azan, accumulation in the vacuole (probably corresponding to what we described as “cavity”). The organ also sinks deeper within the eyestalk tissues. During metamorphosis, because the entire eyestalk rotates in animals of this genus, the organ is displaced to a dorsal position (Hubschman 1963; their Figure 17). Similar complex ontogenetic movements, accompanied by behavioral changes in the animal's orientation while swimming, were reported to take place within the developing eyestalks of *Palaemon serratus* (Bellon‐Humbert et al. 1981). In this species, a larval sensory pore is present in the Zoea 1 and the OB, associated with lateral protocerebrum/terminal medulla, comprises 26 onion bodies. In later larval stages, additional vacuoles develop within the organ (Bellon‐Humbert et al. 1981). Rotllant et al. (1994) described the embryonic development of the OB in the clawed lobster, *Homarus gammarus*. In addition to a pore, numerous onion bodies are present in late embryos of this species, close to the lateral protocerebrum, but no vacuole or cavity exists. This configuration persists into larval stages, when an increasing number of cells, which the authors describe as “vesicular” appear. Taken together, these older studies and our own data suggest that the OB in early larvae of decapod crustaceans comprises a cuticular pore (hence the older name SPX), typical onion bodies with concentric lamellae, and a vacuole/cavity that varies in its presence as key elements.

The functional morphology of the OB was described in many adult malacostracan crustaceans, but its function remains enigmatic. Photoreceptive, chemoreceptive, or baroreceptive sensory functions have been proposed for the adult organ, as well as a mainly secretory function (reviewed in Chaigneau 1994). As shown by Hallberg and Kauri (1992) in *Macrobrachium rosenbergii*, the organ most likely does not have a photoreceptive function, a hypothesis that was already challenged by Chaigneau and Chataigner (1977) on the basis of the observation that the OB is also present in crustaceans living in caves and that is in line with the observation of distinct OBs in crustaceans that live in the deep sea (Charmantier‐Daures and Segonzac 1998). Although crustaceans typically expose their olfactory sensilla to the outside of the body to improve contact with the stimulus (Derby 2021), a potential chemosensory function of the OB cannot be ruled out, perhaps to detect essential molecules such as oxygen or sulfides. Moreover, Chaigneau (1994) suggested considering a function for measuring osmotic pressure. Typical elements of the adult malacostracan OBs are the onion bodies that are extensive ciliated processes linked with sensory cell bodies (Chaigneau 1973). Like for decapod larvae, a close association of the OB with the sensory pore organs is a commonly reported theme in adult crustaceans (Kauri and Lake 1972; Chaigneau 1973; Chaigneau and Besse 1994; Charmantier‐Daures and Segonzac 1998), suggesting that these organs may cooperate in detecting several sensory modalities. It appears that the OBs and associated sensory pore organs represent a presumably multifunctional sensory organ system that, in the past, was neglected by carcinologists who have rather focused on the more obvious crustacean sensory organs such as the CE and paired antennae.

For the present study, Smith's (1974) ultrastructural study of the OB of the European Shore Crab (*C. maenas*) is particularly relevant. This author described the structure of the onion bodies in great detail which in this species consisted of several lobes of concentric membranes, often with numerous granules of varying shape, size, and density at their center and between peripheral membranes. Smith (1974) observed that onion body membranes appeared to be continuous with fields of granules. He also observed onion bodies composed mainly of granules, suggesting a possible secretory function. However, he also reported a modified double ciliary junction between the distal concentric membranes and the associated soma indicating a possible sensory function of the cells. Summarizing his results, Smith (1974) suggested that in *C. maenas*, the OB, which has a distinct axonal connection to the lateral protocerebrum/terminal medulla, may primarily have a neurosecretory function that is controlled by input received from the sensory segment of the onion bodies. Studying the onion bodies of larval brachyurans, such as *H. sanguineus* at an ultrastructural level, will be essential to provide new insights into possible developmental plasticity and perhaps the functions of the onion bodies.

In a series of neurosurgical extirpation experiments of the neurosecretory X‐organ in living *P. serratus*, Bellon‐Humbert et al. (1981) reported that, also in control animals, the onion bodies showed a structural plasticity in that some contained a central vacuole filled with an “undefined” material. The authors interpreted this observed structural plasticity as indicating lysis during neurodegenerative processes. The extirpation experiment had a substantial effect on the OB structure, which varied according to the molt cycle. Bellon‐Humbert et al. (1981) also discussed evidence for seasonal and molt‐cycle‐related structural changes in the OB, as presented by other authors. Immunohistochemistry showed a circadian modulation of serotonin in the OB of *P. serratus* (Bellon‐Humbert and van Herp 1988). These authors also observed a gradient in onion body morphology. In more distal sections of the OB, the onion bodies appeared fully intact, whereas in more proximal sections, they appeared “degenerated” with a vacuole and concentric membranes that had disintegrated into vesicles (Bellon‐Humbert and van Herp 1988, see their Figure 1). In summary, the onion bodies as central OB elements, (Smith 1974), display a high level of structural plasticity depending on season, daytime versus night, and molt cycle (Bellon‐Humbert et al. 1981; Bellon‐Humbert and van Herp 1988). This plasticity may mirror as yet not systematically explored differences in the physiological states of the examined animals. Future studies should also continue to monitor developmental plasticity.

### 4.3 Central Brain, Olfactory Pathway, and Neurochemistry

In adult *H. sanguineus*, the neuroanatomy of the central brain (Tsvileneva and Titova 1985; Tsvileneva et al. 1985) and the localization of several neuroactive substances (Kotsyuba 2012; Kotsyuba and Dyachuk 2021) have been analyzed. The brain architecture in this species is quite similar to that of other brachyuran crabs such as *Scylla serrata* (Sandeman et al. 1992), *C. maenas* (Krieger et al. 2012), *Portunus pelagicus* (Saetan et al. 2013), and several other brachyuran species with different degrees of terrestriality (Krieger et al. 2015). The general arrangement of the protocerebral neuropils, including the PB and the CB in the Zoea 1 of *H. sanguineus*, represents the typical Bauplan seen in the adult brain. However, there are also obvious neuroanatomical differences to adult brains. These resemble similar differences that were observed between the brains of larvae versus adults in *H. araneus* (Harzsch and Dawirs 1996a) and *Pachygrapsus marmoratus* (Geiselbrecht and Melzer 2013). Specifically, with the methods we used, we were unable to detect any distinct anlagen of the following deuto‐ and tritocerebral neuropils that characterize adult crab brains: the median antenna 1 neuropil (receiving input from the statocyst and mechanosensory afferents from sensilla at the base of antenna 1); the lateral antenna 1 neuropil (receiving input from the statocyst and non‐aesthetasc chemoreceptor neurons and mechanoreceptor neurons associated with antenna 1 and is involved in motor control of this appendage); and the antenna 2 neuropil (receiving afferents from antenna 2 and is involved in motor control of this appendage; Sandeman et al. 1992; Mellon 2007, 2012). Although newly hatched crab larvae display gravitaxis (e.g., Park et al. 2004), a statocyst resembling that of adult crustaceans is not present in the larvae, not even as a rudiment (Harzsch, Melzer, unpublished findings). What is more, the number of sensory sensilla on the larval second antennae apparently is so small that their central projections do not make up a distinct neuropil detectable with our methods.

Compared to the brains of, for example, crayfish and spiny lobsters (Sandeman et al. 1992) or hermit crabs (Harzsch and Hansson 2008; Krieger et al. 2012), the deuto‐ and tritocerebrum of brachyuran crabs, in addition to the aforementioned three neuropils, also contain several regions of ill‐defined, diffuse neuropil (“terra incognita”), the structure and connections of which await further exploration. In the Zoea 1 brain of *H. sanguineus*, the seemingly undifferentiated LNP is present in a corresponding position and may later give rise to these adult neuropils mentioned above. More fine‐grained analyses using, for example, backfilling techniques, may reveal the anlagen of these neuropils embedded within the larval LNP. Clearly, as described for the brains of crayfish and clawed lobsters (Helluy et al. 1993, 1995; Mellon and Alones 1993), substantial growth and differentiation characterize the postembryonic development of the crab brain. Using an s‐phase specific proliferation marker, Harzsch and Dawirs (1996a) showed that in the brain of *H. araneus* larvae, new neurons are generated by the mitotic action of asymmetrically dividing stem cells (neuroblasts) that cease dividing during metamorphosis to the Megalopa. Nevertheless, ganglion mother cells continue to divide in the brain of this species until the second metamorphosis to the juvenile Crab 1 so that the complete set of neurons is present by then. Subsequent growth is achieved by axonal and dendritic outgrowth of existing neurons (Harzsch and Dawirs 1996a). A notable exception is the central olfactory pathway where life‐long neurogenesis takes place. This is the case in many decapod crustaceans, including brachyuran crabs (e.g., Beltz et al. 2015, 2016; Beltz and Benton 2017; Brenneis et al. 2021; Benton et al. 2022; reviews Schmidt 2007, 2014; Sandeman et al. 2011).

Another notable difference between larval and adult central brains in *H. sanguineus* concerns the organization of the OL. As in all other decapod crustaceans (reviews, e.g., Sandeman and Mellon 2002; Schachtner et al. 2005; Schmidt 2007; Schmidt and Mellon 2011; Derby and Weissburg 2014; Derby et al. 2016; Schmidt 2016; Harzsch and Krieger 2018), in the brain of adult brachyuran crabs (Sandeman et al. 1992; Krieger et al. 2012, 2015) including *H. sanguineus* (Tsvileneva and Titova 1985; Tsvileneva et al. 1985; Kotsyuba 2012; Kotsyuba and Dyachuk 2021), the synaptic layer that surrounds the fibrous core of the OL is subdivided into distinct subunits of synaptic neuropil, the olfactory glomeruli. There, the axons of olfactory sensory neurons (OSNs) interact synaptically with local olfactory interneurons and projection neurons. Furthermore, it has been well documented that in all decapod crustaceans analyzed to date, the glomeruli are regionalized along their long axis, such that an outer cap, a subcap, and a base region can be distinguished (reviewed in Harzsch and Krieger 2018; and see Harzsch et al. 2022). Such a differentiation is not yet very clearly visible in the Zoea 1, and corresponding findings were obtained by Kotsyuba et al. (2024) in larvae of the anomuran *Paralithodes camtschaticus*. Therefore, in addition to studying the substantial increase in neuropil volume of the OLs during larval life (Harzsch and Dawirs 1996a) in response to the integration of the input from newly generated aesthetascs (Ekerholm and Hallberg 2002), the brain of crab larvae lends itself to analyzing the process of ontogenetic formation of olfactory glomeruli. Such an analysis was conducted by Helluy et al. (1996) in the developing brain of the American lobster *H. americanus* using classical histology. Contemporary immunohistochemical methods combined with confocal laser‐scanning microscopy should allow a more detailed analysis of glomerular development in crustaceans using crab larvae as model systems.

Chemosensory input to the OLs in crustaceans is provided by the axons of OSNs, which are associated with the aesthetasc sensilla on antenna 1 (reviews Hallberg et al. 1992; Hallberg and Skog 2011; Mellon 2014; Derby 2021). In the larvae of *H. sanguineus*, the number of aesthetascs increases from two in the Zoea 1 to nine in the Zoea 5 (Hwang et al. 1993). In comparison, seven aesthetascs are present in the last zoeal stage of *C. maenas* (Ekerholm and Hallberg 2002). In adult brachyuran crabs, the number of aesthetascs per antenna is species‐specific and in aquatic species ranges from 165 in *Percnon gibessi* across 220 in *Xantho poressa* to 285 *C. maenas* and 540 in *C. borealis*. It is below 100 in terrestrial and semiterrestrial crabs (reviewed in Krieger et al. 2015, their Table 2). The numbers of OSNs associated with each aesthetasc were determined for only a few decapod species: about 100 OSNs in the brachyuran crab *Cancer productus*, 175 in the crayfish *P. clarkii*, 350 in the spiny lobster *Panulirus argus*, and 400 in the hermit crab *Pagurus hirsutisculus* (reviewed in Harzsch and Krieger 2018). The number of aesthetascs and associated OSNs is not known in *H. sanguineus*. In a conservative estimate based on a comparison with other aquatic brachyuran crab species, we suggest around 150 aesthetascs, each of which is associated with about 100 OSNs for adult animals *H. sanguineus*. On the basis of this estimate, the chemosensory input to each of the bilaterally symmetric OLs increases from an estimated 200 sensory neurons in the Zoea 1 (with 2 aesthetascs), to 900 in the Zoea 5 (nine aesthetascs) and 15,000 in adult animals.

As summarized in Spitzner et al. (2018; their Table 1) and in Harzsch and Viertel (2020; their Table 1), about a dozen different neuroactive substances have been localized in the embryonic and larval nervous system of crustaceans using immunohistochemistry. In the central brain of brachyuran larvae, the distribution of serotonin (Harzsch and Dawirs 1995) and of RFamide‐like neuropeptides (Harzsch and Dawirs 1996b) so far have been analyzed. Additional information has recently become available on the immunolocalization of dopamine and serotonin in larvae of an anomuran crustacean, the king crab *P. camtschaticus* (Kotsyuba et al. 2024). In the central brain, the Zoea 1 of *H. araneus* (Harzsch and Dawirs 1995) and *H. sanguineus* both show serotonin‐immunoreactivity in the following elements: cell somata in the anteriorly located cell cluster (6), the median protocerebral neuropils, including the CB and commissural neurites that accompany the CB, and commissural neurites at the posterior (tritocerebral) part of the brain just anterior to the esophageal foramen. In both species, the OL is devoid of serotonin‐immunoreactivity in the Zoea 1, although in adult crustaceans this structure displays a rich neurochemistry, including serotonin (see, e.g., Harzsch et al. 2022). The central brain of the Zoea 1 in *H. araneus* and *H. sanguineus* also displays corresponding patterns of RFamide‐like immunoreactivity, with labeling present in the anterior and PPNs, the OLs, the LNP (not labeled as such in Harzsch and Dawirs 1996b), commissural neurites at the posterior (tritocerebral) part of the brain just anterior to the esophageal foramen, and the commissural ganglion. To our knowledge, orcokinin‐like and allatostatin‐like immunoreactivity has not been mapped previously in any brachyuran larvae, but the latter neuropeptide was described to be present in the embryonic lobster pericardial organs and VNC (Pulver and Marder 2002).

### 4.4 Ventral Nerve Cord and Innervation of Neurosecretory Peripheral Release Sites

The development of the crustacean VNC from embryo to adult, including aspects of segmental organization, cell proliferation, and axogenesis, were reviewed by, for example, Harzsch (2003b) and Scholtz (2020). The postembryonic production of new neurons in the VNC of brachyuran larvae is driven by asymmetrically dividing stem cells, the neuroblasts, which give rise to ganglion mother cells that continue to divide (Harzsch and Dawirs 1994). Similar to the brain, the generation of neurons in this species’ VNC seems to be complete at the molt to the Crab 1, so that subsequent growth of the ganglionic neuropil regions is due to the expansion of axonal and dendritic arborizations of existing neurons. In all brachyuran crabs (review Anger 2001), including *H. sanguineus* (Hwang et al. 1993), at hatching, only the appendages associated with the segments of the mandible, maxilla 1 and 2, and maxilliped 1 and 2 are functional. This is despite the Zoea 1 at hatching has already formed all body segments of the brachyuran Bauplan (Harzsch 2003b). Here, we observed that the ganglia within these appendage‐bearing segments are much more elaborate than in more posterior ones. The appendages maxilliped 3, all pereiopods, and all pleopods will gradually develop in successive stages and become functional during the first metamorphosis (summarized in Spitzner et al. 2018). This peculiar aspect of brachyuran development has motivated several studies on neurogenesis in the larval VNC aimed at understanding the relationship of neurogenesis and maturation of the neuromuscular system (Harzsch et al. 1999; Geiselbrecht and Melzer 2013; reviewed in Harzsch 2003b). Geiselbrecht and Melzer (2013) suggested that analyzing larval neurogenesis and, in parallel, appendage development in decapod crustaceans may provide a useful framework for exploring heterochrony, that is, differences in the timing of morphogenetic events. With the latest techniques we have available to study myogenesis and the establishment of neuromuscular connections in crustaceans (review Loose et al. 2020), examining the concerted ontogeny of the VNC and the neuromuscular system in the associated appendages in brachyuran larvae seems a promising enterprise, specifically concerning metamorphosis one (discussed in Spitzner et al. 2018).

The morphology of segmentally arranged, individually identifiable serotonin‐immunoreactive neurons in the VNC of *adult* arthropods has been extensively studied in a phylogenetic context and to extract evolutionary character transformations in diverse groups such as Crustacea (Harzsch and Waloszek 2000), basal Hexapoda (Stemme et al. 2017), Myriapoda (Harzsch 2004; Sombke and Stemme 2017), and Pycnogonida (Brenneis and Scholtz 2015). These neurons are few in numbers and display characteristic morphologies, which recommends these cells as valuable characters for phylogenetic analyses (Harzsch and Waloszek 2000; Stemme and Stern 2015). Serotonin was among the first neuroactive substances that was detected immunohistochemically in the *developing* crustacean VNC, first in developmental stages of the American lobster *H. americanus* (Beltz et al. 1990), then in the larval nerve cord of the spider crab *H. araneus* (Harzsch and Dawirs 1995). These early studies used enzyme‐based secondary detection systems combined with bright field microscopy and provided limited resolution of the neuron's morphology. Nevertheless, equivalents to the lateral and medial serotonin‐immunoreactive cells that we describe here can be found in the images provided in both aforementioned studies, but a one‐to‐one correspondence is difficult to establish. The study by Harzsch (2003a) on the embryonic lobster VNC made use of confocal laser‐scanning microscopy, which provides superior resolution of the neuron's neurite's morphology. A study on larvae of the King Crab, *P. camtschaticus* (Anomura) also reported serotonergic neurons in the larval VNC but not in enough depth of analysis to allow for a meaningful comparison with our data although similar techniques were used (Kotsyuba et al. 2024). In the embryonic lobster VNC, Harzsch (2003a) identified two classes serotonin‐immunoreactive neurons, the anterior (ASC) and posterior (PSC) serotonergic cells and was able to resolve the course of their neurites in great detail. Considering the position of the neuronal somata, the embryonic lobster ASC and PSC correspond to the lateral and medial neurons, respectively, in the larval nerve cord of *H. sanguineus*. Unfortunately, because of a variable labeling quality in our preparations, possibly due to fluctuations of the animal's physiological state during fixation, we were not able to resolve the course of these cell's neurites as an additional morphological criterion for such a comparison, in addition to the soma position. Therefore, future studies that analyze the development of individually identifiable serotonergic neurons across the larval life cycle in brachyuran larvae should use preincubation protocols for living nervous tissue to improve subsequent serotonin immunostaining as proposed by Stemme and Stern (2015) to overcome such methodological shortcomings.

We observed large neurons in the VNC that express RFamide‐like and SIFamide‐like immunoreactivity that innervate peripheral release sites outside the central nervous system. Similar neurons are visible in the larval nerve cord of *H. araneus* (Harzsch and Dawirs 1996b), although this was not recognized by the authors at the time. Comparing our data to the study by Pulver and Marder (2002; their Figures 1 and 6) on the neuropeptidergic innervation of the embryonic pericardial organs in embryos of the American lobster *H. americanus*, we identified these release sites as the “anterior ramifications” based on a similar innervation pattern by neurons showing RFamide‐like immunoreactivity. Like the pericardial organs, the anterior ramifications are characteristic neurosecretory structures in brachyuran crabs, likely associated with the venous sinus system (Maynard 1961a,b; see also Dircksen and Keller 1998, their Figure 2 for *C. maenas*). Our data suggest a close spatial association of the anterior ramifications with the MG which, together with antennal, antennary, and green/renal glands, function as the primary excretory organs of crustaceans (review Freire et al. 2008; and Loose et al. 2020 on ontogenetic aspects). To our knowledge, our study is the first to suggest a close association of the larval anterior ramifications with the MG. Additional studies should examine this association in greater detail to address the question if peripherally released neuropeptides modulate the activity of this excretory organ.

## 5 Conclusions

Decapod larvae can sense a multitude of environmental stimuli such as light, gravity, hydrostatic pressure, tidal currents, temperature, salinity, chemical cues, and food concentration (reviews Cohen and Forward 2009; Forward et al. 2001; Forward 2009; Epifanio and Cohen 2016; Epifanio 2013; Cohen and Epifanio 2020; Morgan 2020), with gravity, hydrostatic pressure, and light considered as the three central external cues affecting larval behavior (review Epifanio and Cohen 2016). In *H. sanguineus*, the influences of gravity and pressure on swimming behavior have been analyzed in newly hatched larvae (Park et al. 2004; Cohen et al. 2015). The role of chemical cues in inducing metamorphosis has been examined in later larval stages (Anderson and Epifanio 2009; Anderson et al. 2010; Anderson and Epifanio 2010). Our neuroanatomical data provide evidence for an elaborate visual system and chemosensory system in the Zoea 1 of *H. sanguineus*. Yet, we failed to locate the larval nauplius eye which in other decapod larvae is located anteriorly embedded within the median protocerebrum (Cronin et al. 2017). Despite behavioral evidence for gravitaxis in newly hatched larvae, we currently lack anatomical evidence for a larval gravity sensor. The typical statocyst, which in adult crabs is located at the base of the second antennae (reviewed in Mellon 2014) remains to develop in the Zoea 1. As for perceiving pressure, the sensory dorsal organs are potential candidates (Laverack et al. 1996). These organs are also present in crab larvae (e.g., Meyer et al. 2006; Lerosey‐Aubril and Meyer 2013; their Figure 2) but we failed to trace these organs in our study. The enigmatic OB, discussed above, provides another, as yet unknown, sensory modality that shapes the larval behavioral repertoire.

We estimated here that in *H. sanguines*, the chemosensory input to each of the bilaterally paired OLs increases from approximately 200 sensory neurons in the Zoea 1 to an estimated 15,000 sensory neurons in adult animals. The input from each CE to the central visual pathway increases from about 900 photoreceptor cells in the Zoea 1 to approximately 46,000 in adults. Although we did not measure any neuropil volumes, it seems that in the Zoea 1, the investment of nervous tissue in the central visual pathway exceeds that of the central olfactory pathway by more than a factor of 5 as would be suggested by comparing the input numbers (200 OSNs vs. 900 photoreceptor neurons). In their book “Principles of Neural Design,” Sterling and Laughlin (2015) suggested that “Visual sensors are fast and energetically costly. Visual processing for form, motion and color is computationally demanding. … The visual system is ‘deep’ and maps spatial position.”, whereas “Olfactory sensors are slow and energetically cheap. The olfactory system is ‘shallow’ and processes globally, without reference to spatial position.” Hence, the question arises whether the differential investment in specific brain structures observed in the larvae mirrors the behavioral relevance of the associated sensory system or rather the computational needs for extracting information from the input? In other words: Is the visual sense compared to the olfactory system so much more critical for the larval behavior as the investment in processing capacity would suggest?

In addition to the well‐developed visual and olfactory organs and related primary visual and olfactory neuropils, the Zoea 1 brain in *H. sanguineus* comprises several characteristic secondary neuropils. These are, for example, the lateral and the median protocerebrum, including the CB. These higher order neuropils in the adult crustacean brain are thought to integrate highly processed multimodal sensory input, shape appropriate behavioral actions, and learn and memorize (reviewed, e.g., in Sandeman et al. 2014). Considering the larval investment in these higher order neuropils, although the lateral protocerebrum is not yet as elaborated as in adult crabs (Strausfeld and Sayre 2021), larvae may be capable of more complex behavioral decisions than previously thought, with potential integration of learning and memory.

### 5.1 Future Lines of Research

From our analysis of the Zoea 1 nervous system in *H. sanguineus*, numerous future research topics arise to be analyzed in subsequent larval stages:

- study the ontogeny of “enigmatic” sensory organs: Organ of Bellonci, dorsal sensory organ, statocyst, and nauplius eye (cf., Cronin et al. 2017)
- analyze the developmental elaboration of the visual satellite neuropilsand mushroom bodies in the eyestalks as well as of the median and lateral antenna 1 neuropils and the antenna 2 neuropil in the central brain
- examine the ontogenetic formation of olfactory glomeruli
- survey the developmental elaboration of the VNC in parallel to the neuromuscular system in the developing thoracic appendages
- determine the relationship of neurosecretory structures (anterior ramifications) and excretory organs

## Author Contributions

Steffen Harzsch conceived the study, performed confocal laser‐scan microscopy and bright field microscopy, 3D reconstruction of CNS with Amira, and scanning electron microscopy, analyzed the data, compiled the figures, and wrote the manuscript. Johanna Blatt dissected the brain and ventral nerve cord, performed immunohistochemistry and fluorescence microscopy; and assisted in drafting the manuscript. Johanna Seegel‐Schultz fixated larvae, dissected the brain, performed immunohistochemistry and confocal laser‐scan microscopy. Lisa Riehemann assisted in larval rearing, dissected the larvae, and performed chemical fixation, and assisted in drafting the manuscript. Noé Espinosa‐Novo assisted in larval rearing and in drafting the manuscript. Jan Phillipp Geißel assisted in larval rearing and in drafting the manuscript. Joshua Gauweiler performed 3D reconstruction of larval eyes with 3D Slicer. Alexandre Casadei‐Ferreira assisted with 3D reconstruction of larval eyes and assisted in drafting the manuscript. Sebastian Büsse supervised 3D reconstruction with Amira and assisted in drafting the manuscript. Birk Rillich digitized the section series. Wolfgang Stein assisted in conceiving the study and edited the manuscript. Gabriela Torres (shared last authorship) provided logistics and coordinated the larval rearing facilities, supervised the larval rearing, and assisted in drafting the manuscript. Roland Melzer (shared last authorship) assisted in analyzing the data, performed light microscopy, and wrote parts of the manuscript.

## Funding

This research was supported by the Marine Stations Helgoland and Sylt operated by the Alfred‐Wegener‐Institut Helmholtz‐Zentrum für Polar‐ und Meeresforschung (Grant No: AWI_BAH_6). This study was also supported by the DFG grant HA2540/20–1, and a Kavli foundation Exploration Award—Neurobiology and Changing Ecosystems, ID LS‐2023‐GR‐47‐2855.

## Conflicts of Interest

The authors declare no conflicts of interest.

## Data Availability

The data that support the findings of this study are available from the corresponding author upon reasonable request.
